# Chemistry domain of applicability evaluation against existing estrogen receptor high-throughput assay-based activity models

**DOI:** 10.3389/ftox.2024.1346767

**Published:** 2024-04-17

**Authors:** Mark D. Nelms, Todor Antonijevic, Caroline Ring, Danni L. Harris, Ronnie Joe Bever, Scott G. Lynn, David Williams, Grace Chappell, Rebecca Boyles, Susan Borghoff, Stephen W. Edwards, Kristan Markey

**Affiliations:** ^1^ RTI International, Research Triangle Park, NC, United States; ^2^ ToxStrategies, Katy, TX, United States; ^3^ ToxStrategies, Austin, TX, United States; ^4^ U. S. Environmental Protection Agency, Washington, DC, United States; ^5^ ToxStrategies, Asheville, NC, United States; ^6^ ToxStrategies, Research Triangle Park, NC, United States

**Keywords:** estrogen receptor, new approach methods, NAMs, chemical prioritization, chemical clustering, endocrine disruptor, endocrine disruptor screening program, EDSP

## Abstract

**Introduction:**

The U. S. Environmental Protection Agency’s Endocrine Disruptor Screening Program (EDSP) Tier 1 assays are used to screen for potential endocrine system–disrupting chemicals. A model integrating data from 16 high-throughput screening assays to predict estrogen receptor (ER) agonism has been proposed as an alternative to some low-throughput Tier 1 assays. Later work demonstrated that as few as four assays could replicate the ER agonism predictions from the full model with 98% sensitivity and 92% specificity. The current study utilized chemical clustering to illustrate the coverage of the EDSP Universe of Chemicals (UoC) tested in the existing ER pathway models and to investigate the utility of chemical clustering to evaluate the screening approach using an existing 4-assay model as a test case. Although the full original assay battery is no longer available, the demonstrated contribution of chemical clustering is broadly applicable to assay sets, chemical inventories, and models, and the data analysis used can also be applied to future evaluation of minimal assay models for consideration in screening.

**Methods:**

Chemical structures were collected for 6,947 substances via the CompTox Chemicals Dashboard from the over 10,000 UoC and grouped based on structural similarity, generating 826 chemical clusters. Of the 1,812 substances run in the original ER model, 1,730 substances had a single, clearly defined structure. The ER model chemicals with a clearly defined structure that were not present in the EDSP UoC were assigned to chemical clusters using a k-nearest neighbors approach, resulting in 557 EDSP UoC clusters containing at least one ER model chemical.

**Results and Discussion:**

Performance of an existing 4-assay model in comparison with the existing full ER agonist model was analyzed as related to chemical clustering. This was a case study, and a similar analysis can be performed with any subset model in which the same chemicals (or subset of chemicals) are screened. Of the 365 clusters containing >1 ER model chemical, 321 did not have any chemicals predicted to be agonists by the full ER agonist model. The best 4-assay subset ER agonist model disagreed with the full ER agonist model by predicting agonist activity for 122 chemicals from 91 of the 321 clusters. There were 44 clusters with at least two chemicals and at least one agonist based upon the full ER agonist model, which allowed accuracy predictions on a per-cluster basis. The accuracy of the best 4-assay subset ER agonist model ranged from 50% to 100% across these 44 clusters, with 32 clusters having accuracy ≥90%. Overall, the best 4-assay subset ER agonist model resulted in 122 false-positive and only 2 false-negative predictions compared with the full ER agonist model. Most false positives (89) were active in only two of the four assays, whereas all but 11 true positive chemicals were active in at least three assays. False positive chemicals also tended to have lower area under the curve (AUC) values, with 110 out of 122 false positives having an AUC value below 0.214, which is lower than 75% of the positives as predicted by the full ER agonist model. Many false positives demonstrated borderline activity. The median AUC value for the 122 false positives from the best 4-assay subset ER agonist model was 0.138, whereas the threshold for an active prediction is 0.1.

**Conclusion:**

Our results show that the existing 4-assay model performs well across a range of structurally diverse chemicals. Although this is a descriptive analysis of previous results, several concepts can be applied to any screening model used in the future. First, the clustering of the chemicals provides a means of ensuring that future screening evaluations consider the broad chemical space represented by the EDSP UoC. The clusters can also assist in prioritizing future chemicals for screening in specific clusters based on the activity of known chemicals in those clusters. The clustering approach can be useful in providing a framework to evaluate which portions of the EDSP UoC chemical space are reliably covered by *in silico* and *in vitro* approaches and where predictions from either method alone or both methods combined are most reliable. The lessons learned from this case study can be easily applied to future evaluations of model applicability and screening to evaluate future datasets.

## 1 Introduction

In 1998, the U.S. Environmental Protection Agency (EPA) established the Endocrine Disruptor Screening Program (EDSP), following the Endocrine Disruptor Screening and Testing Advisory Committee recommendations (EDSTAC, 1998), to prioritize, screen, and test chemicals that potentially interfere with estrogen, androgen, or thyroid hormone–related pathways using a two-tiered battery of low-throughput *in vitro* and *in vivo* assays and tests (U.S. EPA, 1998a; U.S. EPA, 1998b). In 2011, the EDSP published an EDSP for the 21st Century work plan (U.S. EPA, 2011) focused on developing and validating high-throughput (HT) assay batteries to aid in the expeditious screening of chemicals.

EPA presented for peer review and comment (U.S. EPA, 2014) a battery of 18 HT assays coupled with a mechanism-based computational model, termed the ER pathway model, as an alternative for various ER-related Tier 1 screening assays. Judson et al. (2015) described the ER pathway model, which integrates concentration-response data from 18 ER-related HT assays and non-specific assay interference data to make predictions of a chemical’s potential for ER agonism or antagonism (Judson et al., 2015). The ER pathway model was evaluated using *in vitro* data from guideline documents (Judson et al., 2015) and *in vivo* assays (Browne et al., 2015; Kleinstreuer et al., 2016). EPA proposed that the ER pathway model could be a validated alternative for the estrogen receptor (ER) binding, ER transactivation, and rodent uterotrophic assays from the original Tier 1 screening battery (U.S. EPA, 2015). Then, the European Chemicals Agency and European Food Safety Authority together published guidance that the ToxCast ER pathway model provides comprehensive pathway coverage for the biology of the ER signaling pathway (EFSA, 2018). EPA is accepting ToxCast ER pathway model data for 1,812 chemicals as alternatives for EDSP Tier 1 ER binding, ER transactivation, and uterotrophic assays (EFSA, 2018; U.S. EPA, 2023). EPA has worked in an open and transparent manner to establish the scientific basis and robustness associated with the utilization of new approach methods for toxicity testing (U.S. EPA, 2023).

As with any model, it is important to more fully understand the domain of applicability. Here, we have chosen to use the existing ER pathway model and how it relates to the EDSP Universe of Chemicals (UoC) as a case study because of the availability of data and amount of research performed in the development of the existing ER pathway model (Judson et al., 2015). Additionally, it would be useful to be cognizant of whether the existing ER pathway model has differing performance across areas of chemistry in the EDSP UoC (e.g., based on chemical clusters) and how that knowledge may be incorporated into an efficient protocol for screening chemicals. It should be noted that although we are using the existing ER pathway model as a case study, the investigations and learnings are applicable for any future testing regardless of the model.

Chemical category formation has been utilized for decades; for example, to predict the physicochemical properties or toxicological effects of chemicals (Cronin, 2013). National and international regulatory bodies, including the European Union, Canada, the United States, and the Organisation for Economic Co-operation and Development commonly use chemical category approaches (U.S. EPA, 2010; OECD, 2014). The justification behind the chemical category may be based on a variety of characteristics; for example, structural features, physicochemical properties, or a common mechanism, among others.

After clusters have been generated, relevant toxicological (including new approach method or *in vivo*) data can be overlaid onto the chemicals or clusters. From here, the data can be amalgamated with knowledge of the toxicity pathway (e.g., provided by an adverse outcome pathway) to help characterize chemical hazard or risk as part of an integrated testing strategy, tiered testing strategy, or integrated approach to testing and assessment. Alternatively, the available information may be used to guide and/or prioritize chemicals for (further) testing.

Judson et al. (2015) provided predictions of ER agonism and antagonism for 1,812 chemicals and evaluated the results based on a set of 45 positive and negative reference chemicals. These included 28 agonist positives, 12 agonist negatives, 4 antagonist positives, and 14 antagonist negatives, with some chemicals being both agonist and antagonist reference chemicals (OECD, 2012; Browne et al., 2015; Kleinstreuer et al., 2016). The agonist portion of the existing full ER pathway model (herein referred to as the full ER agonist model), utilizing a 16-assay subset of the original pathway model, was compared against *in vitro* reference chemicals and *in vivo* uterotrophic studies (Browne et al., 2015). When activity in the full ER agonist model was defined as an area under the curve (AUC) ≥0.1, the accuracy was 93% for predicting *in vitro* assay reference chemicals (i.e., activity from ER binding and ER transcriptional activation assays) and 86% for *in vivo* assay reference chemicals (i.e., guideline-like uterotrophic studies). A separate study, using the full ER agonist model, was conducted to identify the minimum subset of assays that could predict potential ER agonist activity with performance similar to that of the full ER pathway model (Judson et al., 2017). This study was based on a subset of 1,811 chemicals and demonstrated that as few as four assays are needed to predict a chemical’s ER agonism potential with performance similar to that of the full ER pathway model. To identify this minimal set, Judson et al. (2017) used data from the 16 ER agonism-related HT assays present in the full ER agonist model to develop a total of 65,535 ER agonist subset models: one for each combination of 1–16 *in vitro* assays.

The previously chosen best 4-assay system includes an assay (NVS_NR_hER) that is no longer available. However, it may be possible to find or develop an assay that performs similarly to replace it. Alternatively, another minimal assay ER pathway model could be developed. This article demonstrates a method of comparing the performance of any minimal assay model with data with the full model based on structural clusters. This article also proposes how this granular knowledge of the performance of a minimal assay set could be informative for developing the most efficient method to screen chemicals.

The present study has several objectives, including determining whether there are structural classes of chemicals in the EDSP UoC for which the performance of a minimal assay ER agonist model (using the existing best 4-assay ER agonist subset model as a case study) is not as predictive of estrogen agonist activity compared with the existing full ER pathway model. To address this issue, the EDSP UoC was organized into clusters based on chemical-structural features, and the substances that have been tested by the ToxCast/Tox21 program (Collins et al., 2008; Richard et al., 2016; Richard et al., 2021) were mapped into these clusters using the k-nearest neighbors (KNN) classification algorithm. This study also attempts to evaluate the coverage of the EDSP UoC clusters against the 1,800-plus substances used to develop the full ER agonist model (Judson et al., 2015). Finally, this study evaluates the performance of the existing best 4-assay ER agonist subset model (Judson et al., 2017) relative to the existing full ER agonist model and structural clusters to investigate the potential causes of low performance. Although this particular 4-assay ER agonist subset model cannot be performed in the future because one of the assays is no longer available, it is possible that a similar model could be developed, or this analysis could be performed similarly on data generated with another set of ER assays. Although consideration of chemical clustering could benefit the accuracy of ER agonist predictions from the existing full and subset models, the focus of this study is not to develop a method of predicting a chemical’s potential for ER agonism. This work proposes a more robust analytical framework for integrating the adequacy of screening and validation across the chemical space particularly the considerations when building reduced assay sets against more redundant models. It explicitly includes chemical structure and chemical structure diversity overlaid onto assay space to assess the confidence of biological responses to high-throughput assays and computational models. This analysis can be used to additionally assess the adequacy of data and the domain of applicability for building QSAR models from a chemical space perspective.

## 2 Methods

The code for all analyses described below can be found at: https://github.com/USEPA/edsp-er-subset-model-analysis.

### 2.1 Data sources

Four data sources were used in this study: the 2012 EDSP UoC, ToxCast/Tox21 HT screening data, existing full ER pathway and ER agonist model data from Judson et al. (2015), Judson et al. (2017), and EPA’s CompTox Chemicals Dashboard.

#### 2.1.1 EDSP UoC

The EDSP UoC is a list of approximately 10,200 substances, as defined under the Federal Food, Drug, and Cosmetic Act and the Safe Drinking Water Act 1996 amendments. To facilitate the analysis, the EPA authors provided a computable version of the published EDSP UoC (U.S. EPA, 2012).^1^

#### 2.1.2 ToxCast/Tox21 HT screening data

The ToxCast/Tox21 HT screening data consists of approximately 9,500 substances covering a wide variety of chemical uses (including, but not limited to, pharmaceuticals, pesticides, insecticides, and surfactants), which have been tested in approximately 1,400 assays as part of the ToxCast/Tox21 program. Information pertaining to whether a chemical was tested as part of the ToxCast/Tox21 program was downloaded as a comma-separated values file as part of EPA’s invitrodb v3.2 (U.S. EPA, 2020). Meanwhile, assay results were extracted from invitrodb v3.3 using the tcpl R package (v2.0) (Filer et al., 2017). Assay data extracted included the chemical assay AC50 values, the binary hit call (i.e., representing activity [1] or inactivity [0] within a given assay), and the chemical-specific cytotoxicity point.

#### 2.1.3 Full ER pathway and ER agonist model data in the form of AUC scores

The existing full and subset ER pathway models consist of 1,812 chemicals from ToxCast/Tox21 that have been tested in 18 ER-related HT *in vitro* assays.^2^ The results from the existing full ER pathway model (Judson et al., 2015) and the existing full and subset ER agonist models (Judson et al., 2017) were downloaded, as Excel files, from the Supplementary Material sections of the respective journal articles. In this study, we chose to use the existing subset ER model identified as the “best” 4-assay subset model from Judson et al. (2017) as our comparator. This existing 4-assay subset ER agonist model consists of a human cell-free binding assay (NVS_NR_hER), a protein dimerization assay (OT_ER_ERaERb_1440), a reporter assay (ATG_ERa_TRANS), and a proliferation assay (ACEA_T47D_80hr_Positive) and was identified in (Judson et al., 2017) because it had the highest minimum balanced accuracy while using the fewest assays. Throughout this study, we will refer to this subset ER agonist model as the best 4-assay subset ER agonist model.

#### 2.1.4 EPA’s CompTox Chemicals Dashboard

EPA’s CompTox Chemicals Dashboard contains a variety of information for approximately 875,000 chemicals. Defined chemical structures were needed to perform the data analyses in this study. As such, simplified molecular-input line-entry system (SMILES) strings and quantitative structure activity relationship (QSAR)-ready SMILES strings were extracted via a batch search of the CompTox Chemicals Dashboard (Williams et al., 2017).

In addition to the structure information, we extracted Distributed Structure-Searchable Toxicity (DSSTox) substance identifiers (DTXSIDs), DSSTox chemical identifiers (DTXCIDs), Chemical Abstracts Service registry numbers (CASRNs), and preferred chemical names.

To retrieve this information for the EDSP UoC substances, the name and CASRN of each substance were utilized as the inputs to the batch search feature. The search was conducted on both name and CASRN to assist with the manual quality check undertaken on the EDSP UoC. A further search of the CompTox Chemicals Dashboard was conducted to find the chemical components for those substances with a DTXSID and identified as either a substance with a Markush structural representation or of unknown or variable composition, complex reaction products, or biological materials (UVCB; throughout this study, UVCB will be used to represent all complex chemical mixtures). Meanwhile, the DTXSID or CASRN was used as the batch search input for the ToxCast/Tox21 and ER model substances, respectively.

### 2.2 Creation of structural fingerprints

The ToxPrint chemotype feature set (v2.0_r711, https://toxprint.org) and ChemoTyper software (https://chemotyper.org/) were used to create ToxPrint fingerprints. The ToxPrint chemotypes are a predefined library of 729 sub-structural features designed to encapsulate a broad range of chemical atoms and scaffolds (Yang et al., 2015).

Binary molecular fingerprints were created using the SMILES strings for each substance (or known chemical component of a UVCB material) in the EDSP UoC and ER model datasets. These are hereafter referred to as SMILES-based ToxPrint fingerprints.

### 2.3 Creation of an EDSP database

After the chemical structure information was retrieved from the CompTox Chemicals Dashboard (see Section 2.1), a manual quality check of the EDSP UoC was performed to identify potential duplicate entries and instances where the search results of the substance name and CASRN did not match the same DTXSID (in this case, both substances were added).

### 2.4 Data analysis

Unless otherwise stated, all analyses in this study were performed using R, v3.6.3 (R. Core Team, 2019).

### 2.5 Chemical clustering

As we were wanting to create structurally similar chemical clusters for the EDSP UoC and understand how the ER model chemicals fit into these clusters, we first clustered the EDSP UoC, then incorporated the ER model chemicals into the EDSP UoC clusters. The SMILES-based ToxPrint fingerprints were used to perform the clustering. This was done so that as many of the chemicals in the EDSP UoC as possible could be clustered.

To generate the EDSP UoC clusters, we started by calculating the pairwise distances between each of the chemicals and UVCB chemical components in the EDSP UoC using the SMILES-based ToxPrint fingerprints and the Tanimoto distance [i.e., 1—Tanimoto coefficient, as implemented within the philentropy R package [v0.4.0] (Drost, 2018)]. The SMILES-based ToxPrint fingerprints were used here so that all of the ToxPrint structural features present in the chemicals would be considered when generating the clusters. The final chemical clusters were created using hierarchical clustering implementing Ward’s algorithm and a cut height of 1.

Once the EDSP UoC chemicals were clustered, the ER model chemicals were assigned to one of the EDSP UoC clusters using the k-nearest neighbors (KNN) classification algorithm. To ensure consistency in the features considered, we used the SMILES-based ToxPrint fingerprints of the ER model chemicals. To identify the most appropriate value of *k* to use when assigning the ER model chemicals to an EDSP UoC cluster, we split the clustered EDSP UoC chemicals into “training” and “test” sets comprising 80% and 20% of the data, respectively. Although there is no empirically “true” Cluster ID for the EDSP UoC (because it will change depending on the cut height chosen), we determined our most appropriate cut height; thus, we can use the final Cluster ID to provide a “known” Cluster ID for our purposes. The KNN algorithm was then used to predict the Cluster ID of the test set chemicals based upon the *k* nearest chemicals in the training set, changing the value of *k* each time: values of *k* used were between 1 and 5. After each value of *k*, the predicted Cluster IDs for each chemical in the test set were compared against the “known” Cluster ID for the same chemical generated by the hierarchical clustering with a cut height of 1 and the accuracy across all predictions was calculated. The value of *k* with the best accuracy was utilized to assign the ToxCast/Tox21 chemicals to one of the EDSP clusters. Here, based on the test set results, the most accurate value of *k* and the value used in this study was *k* = 1.

### 2.6 Comparing best 4-assay subset ER agonist model results with full ER agonist model results

To determine whether there are clusters where the existing full ER agonist model works well, but the existing best 4-assay subset ER agonist model does not, the results from the best 4-assay subset ER agonist models were compared against the ER agonist results from the full ER agonist model for each cluster. To achieve this, for each chemical in the ER model set with a SMILES string, the ER agonist AUC prediction from the best 4-assay subset ER agonist model was calculated using the median chemical-assay AUC value and assay weights for the relevant subset model [present in the Supplementary Material of (Judson et al., 2017)]. The best 4-assay subset ER agonist model AUC values were then merged with the ER agonist AUC prediction from the full ER agonist model [present in the Supplementary Material of Judson et al. (2017)]. This process was also conducted using the lower and upper 95% confidence interval chemical-assay AUC values from Judson et al. (2017) to calculate the 95% confidence interval AUCs for each subset model. The AUC value predictions for both the full ER agonist model and the best 4-assay subset ER agonist model were, subsequently, dichotomized based upon the threshold value identified in Judson et al. (2017): chemicals with an AUC value ≥ 0.1 were identified as active for ER agonism (i.e., assigned a value of 1), and chemicals with an AUC value < 0.1 were identified as inactive for ER agonism (i.e., assigned a value of 0).

Cluster-level statistics were calculated by comparing the best 4-assay subset ER agonist model and full ER agonist model predictions for clusters containing two or more chemicals in the ER agonist model dataset. The cluster-level statistics calculated include 1) the mean best 4-assay subset ER agonist model AUC, 2) the mean full ER agonist model AUC, 3) the mean difference between the best 4-assay subset ER agonist model AUC and full ER agonist model AUC [calculated as mean (subset ER agonist model AUC—full ER agonist model AUC)], and 4) the standard deviation in the difference between best 4-assay subset ER agonist model AUC and full ER agonist model AUC.

Meanwhile, for the dichotomized predictions, a 2 × 2 confusion matrix was generated for each cluster, from which statistics including sensitivity, specificity, accuracy, balanced accuracy, positive predictive value, and negative predictive value were calculated. In this study, we are using the full ER agonist model results as our “truth.” As such, true positives will be chemicals predicted as agonists by both the full ER agonist model and the best 4-assay subset ER agonist model, true negatives will be chemicals not predicted as agonists by both the full ER agonist model and the best 4-assay subset ER agonist model, false positives will be chemicals not predicted as ER agonists by the full ER agonist model but predicted as ER agonists by the best 4-assay subset ER agonist model, and false negatives will be chemicals predicted as ER agonists by the full ER agonist model but not predicted as ER agonists by the best 4-assay subset ER agonist model.

## 3 Results and discussion

### 3.1 EDSP UoC and ER model chemicals

As can be seen in Table 1, the EDSP UoC contains a total of 10,272 substances. For 6,947 of these substances, we retrieved SMILES strings from the CompTox Chemicals Dashboard. However, it should be noted that this value does not include the EDSP substances that are known mixtures for which SMILES strings were obtained for the chemical components or those substances with a Markush representation. Furthermore, we were able to retrieve QSAR-ready SMILES strings for 6,172 EDSP substances. Unless otherwise stated, all results discussed in this study will focus on those substances for which SMILES strings were available.

**TABLE 1:** The total number of substances within the EDSP UoC, the ToxCast/Tox21 program, and the ER model set, alongside the number of substances for which SMILES and QSAR-ready SMILES were retrieved from EPA’s CompTox Chemicals Dashboard (not including UVCBs, mixtures, or chemicals with Markush representations).

Data source	Total substances	Chemicals with SMILES	Chemicals with QSAR-ready SMILES
EDSP UoC	10,272	6,947	6,172
ER model	1,812	1,730	1,703

Of the 6,947 EDSP UoC substances with a SMILES string, 1,385 are also present in the ER agonist model chemical list and have, therefore, been tested in all 16 ToxCast/Tox21 ER agonist assays that comprise the ER pathway models.

### 3.2 Chemical clustering of EDSP UoC

Using Ward’s hierarchical clustering algorithm and the cut height of 1, the EDSP substances with SMILES-based ToxPrints fingerprints were clustered into a total of 826 clusters (Supplementary Data S1). The cut height of 1 was chosen to balance competing priorities: exclude the generation of clusters containing only a single chemical and limit the number of clusters containing only two chemicals, while not setting the cut height too high so that obviously dissimilar chemicals were clustered together.

### 3.3 Mapping ER model chemicals to EDSP UoC clusters

Using the KNN algorithm, 1,730 ER model chemicals with SMILES-based ToxPrint fingerprints were assigned to one of 557 EDSP UoC clusters (Supplementary Data S2). This assignment of chemicals to clusters included 1,385 ER model chemicals with SMILES-based ToxPrint fingerprints that were also contained within the EDSP UoC. As we would expect, all 1,385 chemicals present in both datasets were assigned by the KNN mapping to the same cluster as the EDSP UoC chemical.

### 3.4 Best 4-assay subset ER agonist model versus full ER agonist model results

The results of the existing best 4-assay subset ER agonist model [identified in Judson et al. (2017)] were compared against those of the existing full ER agonist model on a cluster-by-cluster basis. This task was undertaken to identify whether there are clusters for which the best 4-assay subset ER agonist model does not perform quite as well as the full ER agonist model. We were able to retrieve structural information in the form of SMILES strings for 1,730 of the 1,811 chemicals present in the ER model dataset. Using the KNN algorithm and workflow described earlier, these chemicals were assigned to 1 of 557 EDSP UoC clusters. The vast majority of the 557 clusters contain only chemicals predicted to be negative for ER agonism, irrespective of whether the full ER agonist model or the best 4-assay subset ER agonist model was used.

When considering the full ER agonist model, a total of 503 clusters (90%) contained only chemicals predicted not to be ER agonists. Meanwhile, this number was reduced to 432 clusters (78%) where both the full ER model and the best 4-assay subset ER agonist model predicted no agonist activity. Conversely, 71 clusters contained only chemicals with a negative prediction for ER agonism in the full ER agonist model and one or more chemicals with a positive prediction in the best 4-assay subset ER agonist model. These 71 clusters were composed of 287 ER model chemicals, of which 88 had discrepant results. The median AUC prediction for the full ER agonist model across these 88 chemicals was relatively low at 0.021, compared with the median AUC prediction for these same chemicals in the best 4-assay subset ER agonist model, which was just above the 0.1 threshold for an active prediction at 0.132. Six chemicals had an AUC prediction >0.2 in the best 4-assay subset ER agonist model. Of these six chemicals, the three with the largest AUCs in the best 4-assay subset ER agonist model were all from the same cluster (Cluster 419) and are known ER antagonists (i.e., tamoxifen [DTXSID1034187], AUC = 0.338; tamoxifen citrate [DTXSID8021301], AUC = 0.328; and clomiphene citrate [DTXSID8020337], AUC = 0.277). Two of the remaining three chemicals (clorophene [DTXSID5020154], AUC = 0.237; and 4-chloro-3-methylphenol [DTXSID4021717], AUC = 0.206) were also from the same cluster (Cluster 462). There is some conflicting evidence within the literature that these two chemicals may act as ER agonists *in vitro* (Houtman et al., 2004; Schmitt et al., 2012; Kenda et al., 2020). Some evidence suggests that 4-chloro-3-methylphenol may have estrogenic and/or anti-androgenic effects *in vivo*, with the lowest-observed-adverse-effect level being based upon changes in male reproductive endpoints and changes in the sex ratio at the mid-level of two studies (Superfund Health Risk Technical Support Center, 2009). These chemicals also exhibit activity across the ToxCast/Tox21 assays related to ER receptor binding, dimerization, and RNA transcription in the full ER agonist model; however, neither chemical is active in the assays related to DNA binding, protein production, or ER-induced proliferation. Based on the activity across multiple stages of the full ER pathway model, conflicting *in vitro* evidence, and (current) lack of *in vivo* data, hypotheses could be made that these chemicals may be either partial or (very) weak ER agonists. Meanwhile, two clusters each contained one ER model chemical with a positive prediction for ER agonism in the full ER agonist model and a negative prediction in the best 4-assay subset ER agonist model. The AUC prediction in the best 4-assay subset ER agonist model was just below the 0.1 threshold for chloromethyl methyl ether (DTXSID6020307) and colforsin (DTXSID8040484) at 0.0976 and 0.0901, respectively (i.e., borderline negatives).

When comparing the binary predictions made by the best 4-assay subset ER agonist model against the full ER agonist model across all 1,730 chemicals, 124 differed in their predictions. Almost all inaccurate predictions (122 out of 124) were false positives; that is, the best 4-assay subset ER agonist model indicated the chemical as positive for ER agonism, whereas the full ER agonist model indicated the chemical as negative. Of the chemicals with a false positive prediction, 12 were predicted to be ER antagonists according to the full ER pathway model; this relates to 66.67% of the total number of predicted ER antagonists (18 chemicals) from the full ER pathway model. It should be noted that chemicals identified as antagonists by the full ER pathway model tend to also have agonist AUC values greater than 0.1, but the antagonist AUC is larger than the agonist AUC. The ER antagonist AUC values from the full ER pathway model are larger than the AUC values from the best 4-assay subset ER agonist model for all but 2 of these 12 chemicals. The median AUC value for the 122 false positives from the best 4-assay subset ER agonist model was 0.138, whereas the threshold for an active prediction is 0.1. In comparison, the median AUC from the full ER agonist model for the same set of chemicals was 0.024. These results are similar to those observed by Judson et al. (2017). Whereby, on average, for chemicals with an AUC close to 0 in the full ER pathway model, all subset ER agonist models tended to over-predict the full ER pathway model AUC values. However, these over-predictions appear to be relatively small.

Judson et al. (2017) attributed this tendency for over-prediction to the fact that the full ER pathway model has additional assay interference pathways (termed “pseudoreceptors”) into which these chemicals likely would have been moved (Judson et al., 2015; Judson et al., 2017). These pseudoreceptors are not present in the subset ER agonist models. Another reason for the larger proportion of false positives may be due to the smaller number of assays present in the best 4-assay subset ER agonist model being less able to compensate for strong (potentially) erroneous activity in one assay (or moderate activity in multiple assays) compared with the full ER agonist model. Similarly, the two chemicals with false-negative predictions (chloromethyl methyl ether and colforsin) have a full ER agonist model AUC value slightly or somewhat above the 0.1 threshold for an active prediction (0.2 and 0.1, respectively), whereas the AUC for the best 4-assay subset ER agonist model is just below the 0.1 threshold (0.098 and 0.09, respectively).

**FIGURE 1:**
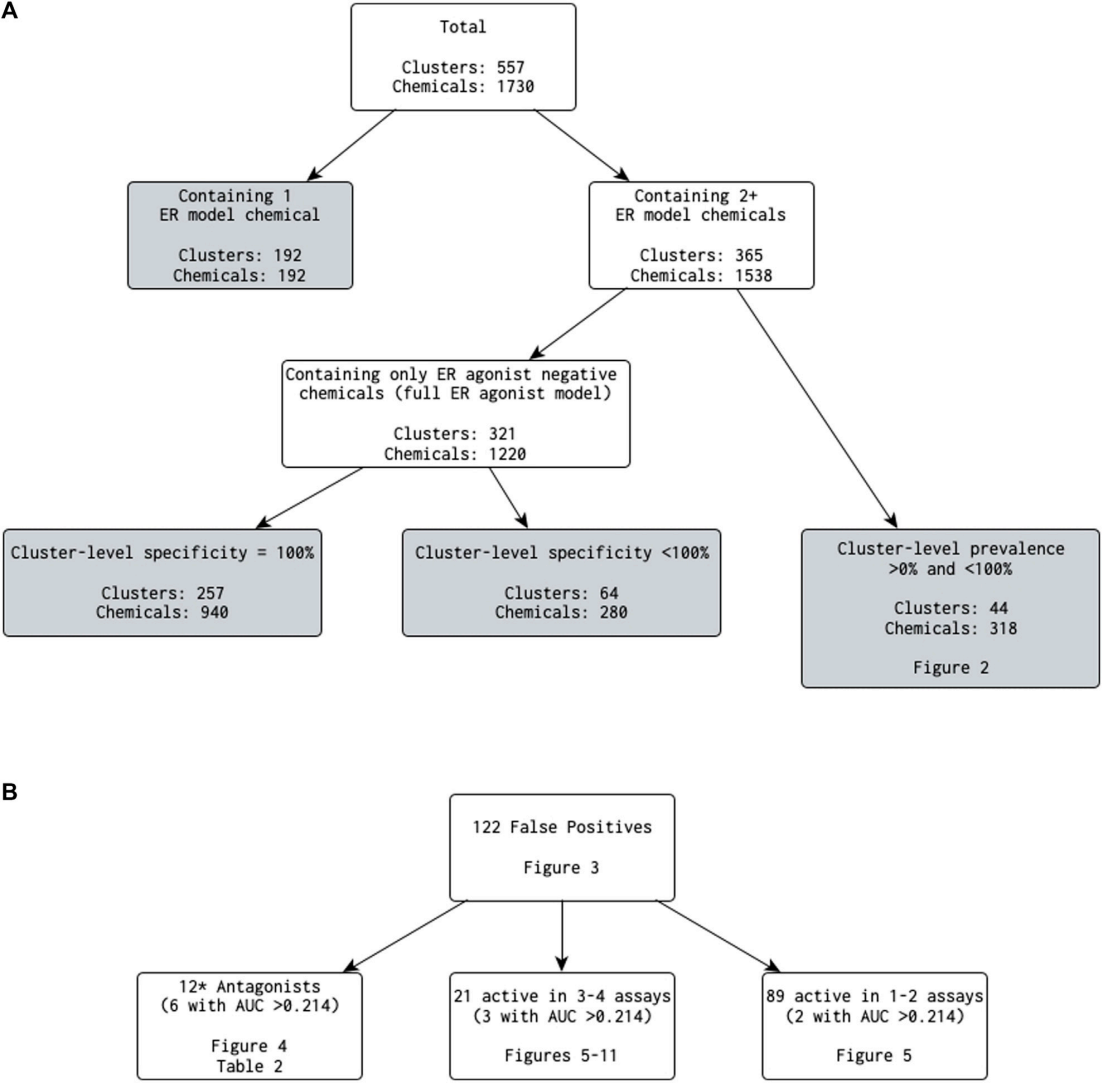
**(A)** Breakdown of the number of clusters (and chemicals contained within) that meet each criterion when investigating results comparing the best 4-assay subset ER agonist model with the full ER agonist model **(B)** Categories of false-positive predictions. NB: 0.214 is the lower 25th percentile AUC from the best 4-assay subset ER agonist model for the true-positive chemicals. *The antagonist count includes one chemical that was in a cluster by itself, which is not discussed in the main text.

Next, we removed clusters containing only one ER model chemical and calculated various statistics on a per-cluster basis (Figure 1). A total of 192 clusters (34.5%) contained only a single ER model chemical. Removing these clusters reduced the number of clusters under investigation to 365, covering 1,538 ER model chemicals, of which 1,222 are also in the EDSP UoC. Of these 365 clusters, 321 clusters (covering 1,220 ER model chemicals, including 994 in the EDSP UoC) were composed of only chemicals predicted by the full ER agonist model as inactive for ER agonism (i.e., a cluster-level prevalence of 0%). The best 4-assay subset ER agonist model predictions exactly matched those of the full ER agonist model for 257 of the 321 clusters (covering 940 ER model chemicals, including 783 in the EDSP UoC). The remaining 64 clusters (covering 280 ER model chemicals, including 211 in the EDSP UoC) had a cluster-level specificity of less than 100% (i.e., at least one chemical in the cluster was predicted by the best 4-assay subset ER agonist model to be a false-positive ER agonist). This corresponds to 81 false-positive chemicals (including 65 in the EDSP UoC), which will be evaluated in more detail as follows.

**FIGURE 2:**
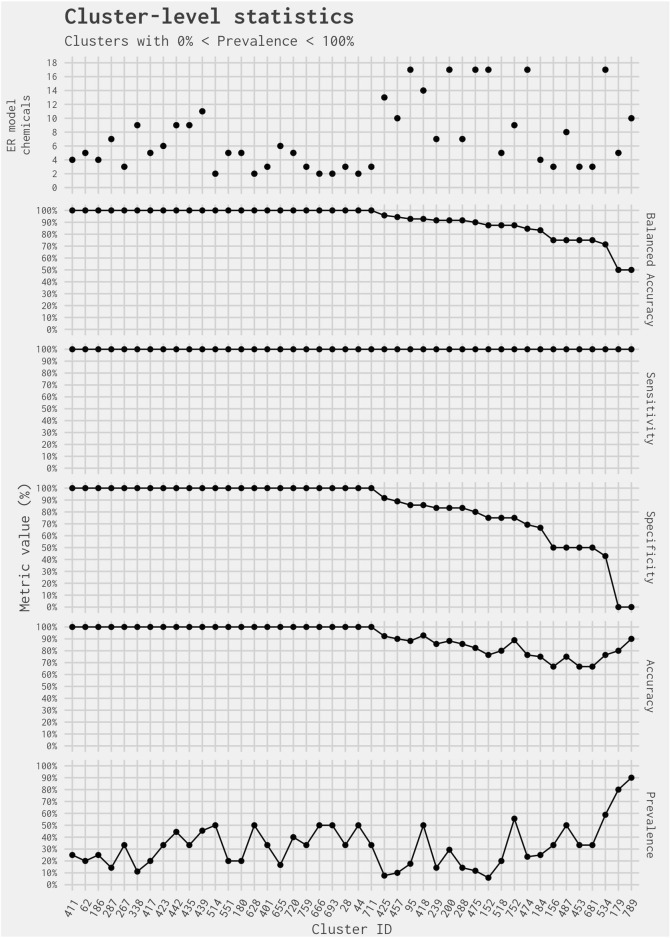
Prediction metrics comparing the best 4-assay subset ER agonist model against the full ER agonist model on a per-cluster basis. Clusters included contain more than one ER model chemical and have prevalence between 0% and 100%. Note that the small number of chemicals in the cluster results in large drops in specificity and balanced accuracy due to a single chemical. As a result, the actual values should not be compared against typical results found in larger datasets.

The remaining 44 of 365 clusters (covering 318 chemicals, including 228 in the EDSP UoC) have a cluster-level prevalence greater than 0% and less than 100% (i.e., containing at least one chemical predicted as active and at least one chemical predicted as inactive by the full ER agonist model) (Figure 1). Out of the 318 ER model chemicals included in the 44 clusters, there are no false-negative chemicals and 34 false-positive chemicals. Figure 2 illustrates the cluster-level balanced accuracy, sensitivity, specificity, accuracy, and prevalence for this set of 44 clusters. The cluster-level balanced accuracy across these 44 clusters ranges from 50% to 100%, with 32 clusters (72.7%) having a balanced accuracy ≥90%. The mean cluster-level balanced accuracy is 91.9%. This is very close to the minimum balanced accuracy for this same subset model across all chemicals in Judson et al. (2017). The decreases in cluster-level balanced accuracy are driven by decreases in specificity, with 100% sensitivity seen across all clusters (Figure 2). This is not surprising because there are only two false-negative chemicals in the entire dataset.

The two clusters with the lowest balanced accuracy and specificity (Clusters 179 and 789) each contain only one chemical predicted as inactive for ER agonism in the full ER agonist model: 4-hexylresorcinol in Cluster 179 and fulvestrant in Cluster 789. In both instances, the chemical is predicted as active for ER agonism in the best 4-assay subset ER agonist model. However, it is less of a surprise that fulvestrant [a known ER antagonist and ER antagonist reference chemical in Judson et al. (2015)] was picked up as an ER agonist because the best 4-assay ER agonist subset model does not simultaneously screen for ER antagonism. Both clusters illustrate the difficulty in interpreting balanced accuracy when looking at individual clusters. Given their small size (and, therefore, limited number of “real” positives and negatives), if one chemical is incorrectly predicted, the statistics for the cluster can seem much worse than the reality: this appears to be the case for Clusters 179 and 789. In each instance, the cluster-level balanced accuracy is inferring that the performance of the best 4-assay subset ER agonist model is poor. However, the balanced accuracy is affected by the fact that there is only one inactive chemical in the cluster, and the prediction for that chemical was incorrect. In contrast, the cluster-level accuracy and sensitivity values for these two clusters are much greater. The cluster-level accuracy is 80% for Cluster 179% and 90% for Cluster 789; meanwhile, the cluster-level sensitivity for both clusters is 100%. More important than the cluster-level statistics, however, is whether there are structural features associated with a false-positive call from the best 4-assay subset ER agonist model. This could allow a combination of biological activity and structural information to be used when selecting chemicals for further screening.

### 3.5 Investigating clusters containing false-positive predictions

Although the number of false-positive chemicals (122) is relatively low compared with the number of chemicals evaluated using each model (1,730), we wanted to find out whether it would be possible to discern criteria that may be utilized as part of a screening strategy to further limit unnecessary testing. To do this, we further investigated the clusters containing one or more false-positive predictions (based on the dichotomized AUC values) (Supplementary Data S3). A total of 91 clusters contained at least one chemical with a false-positive prediction: seven clusters were composed of only a single ER model chemical. Although seven clusters could not be examined further in terms of comparing the results from the false-positive chemical against other chemicals in the same cluster, the chemicals were still of use when analyzing across all chemicals with a false-positive prediction and so were retained.

**FIGURE 3:**
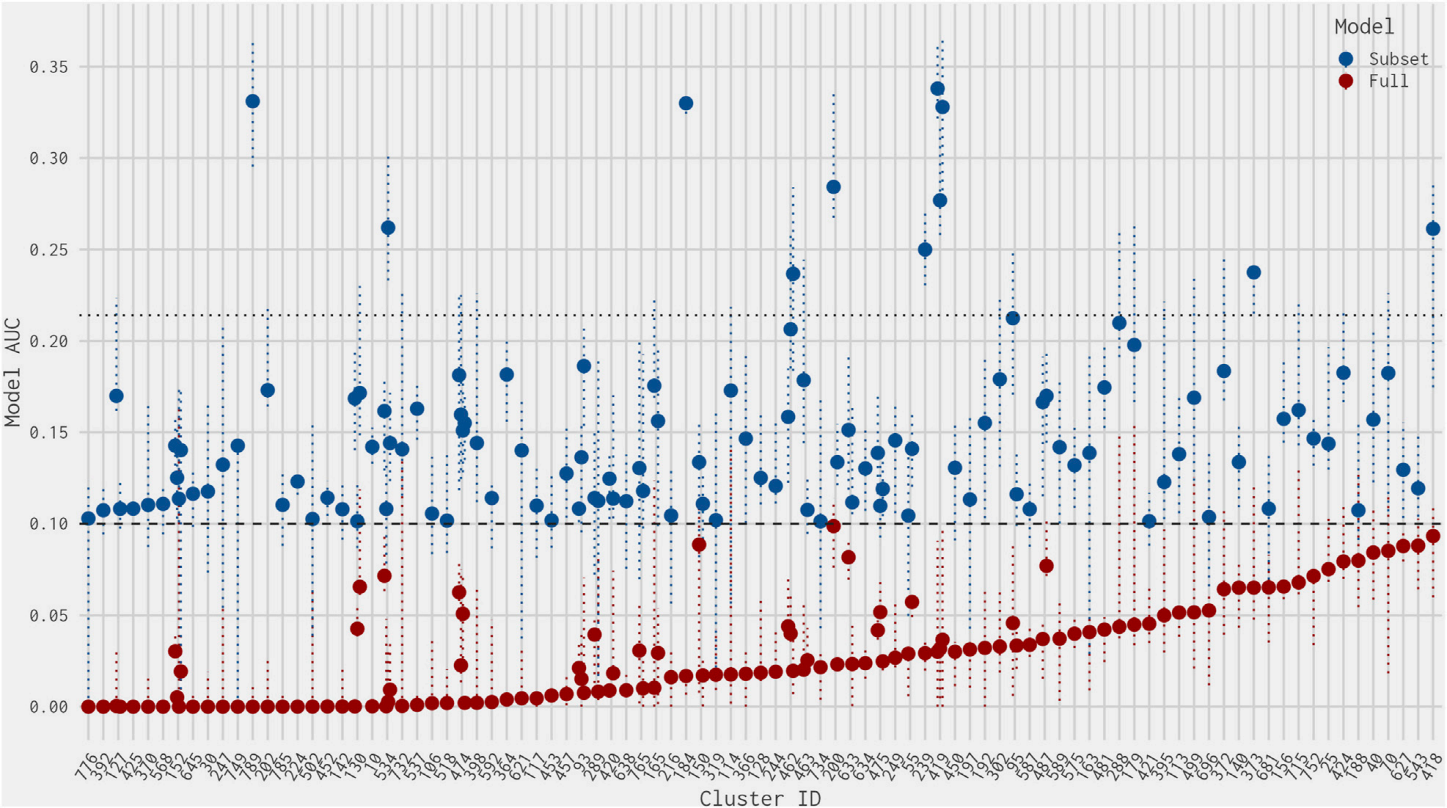
Dot plot of AUC values from the best 4-assay subset ER agonist model (blue) and the full ER agonist model (red) for each chemical predicted to be a false positive (ordered by smallest full ER agonist model AUC) per chemical cluster. The dotted horizontal line at 0.1 represents the threshold for an ER agonist prediction. The dotted horizontal line at 0.214 represents the lower quartile of the best 4-assay subset ER agonist AUC values of the true-positive chemicals. The vertical dotted colored lines represent the upper and lower 95% confidence intervals of the AUC predictions for the full ER agonist model (red) and the best 4-assay subset ER agonist model (blue).

Figure 3 illustrates the AUC values from the full ER agonist model and the best 4-assay subset ER agonist model for all chemicals with a false-positive prediction within each chemical cluster (the distribution of all AUC values for the best 4-assay subset ER agonist model per outcome can be seen in Supplementary Figure S1). Roughly half (64 out of 122, 52.5%) of the false-positive chemicals have 95% confidence intervals for their AUC predictions that overlap with the 0.1 threshold in either the full ER agonist model or the best 4-assay subset ER agonist model (Watt and Judson, 2018). This makes it more difficult to ascertain whether these are “real” false-positive predictions. The majority (90.1%) of chemicals with a false-positive prediction have an AUC value in the best 4-assay subset ER agonist model of less than 0.214, which is the lower 25th percentile AUC of the true-positive chemicals. As such, a prioritization process based solely upon the AUC values would mean that 75% of the true-positive chemicals would likely be tested further before roughly 90% of chemicals with a false-positive prediction. This is discussed in more detail in Sections 3.6 and 4. Meanwhile, only 11 false-positive chemicals out of the 122 false positives have an AUC in the best 4-assay subset ER agonist model equal to or greater than 0.214 (Table 2). These 11 chemicals belong to nine separate chemical clusters, with only one cluster (Cluster 419) containing multiple chemicals with an AUC greater than 0.214. Examining these 11 chemicals further, we found that 6 of the 11 are predicted to be ER antagonists in the full ER pathway model present in Judson et al. (2015) (Table 2). Out of the top 11 chemicals, only 2-naphthalenol has a subset model AUC value larger than the AUC values of any of the chemicals with an antagonist prediction.

**TABLE 2:** The 11 chemicals with a false-positive prediction and an AUC value in the best 4-assay subset ER agonist model greater than 0.214 (i.e., the lower 25th percentile AUC value of the chemicals with a true-positive prediction).

DTXSID	Name	Predicted cluster	Full model AUC	Subset model AUC	2 2015 max receptor	Number of active assays
DTXSID1034187	Tamoxifen	419	0.030	0.338	Antagonist	3
DTXSID4022369	Fulvestrant	789	0.000	0.331	Antagonist	3
DTXSID3037094	4-Hydroxytamoxifen	184	0.017	0.330	Antagonist	2
DTXSID8021301	Tamoxifen citrate	419	0.037	0.328	Antagonist	3
DTXSID5027061	2-Naphthalenol	200	0.099	0.284	None	3
DTXSID8020337	Clomiphene citrate (1:1)	419	0.032	0.277	Antagonist	2
DTXSID5023322	Mifepristone	534	0.003	0.262	Antagonist	3
DTXSID3040776	Morin hydrate	418	0.093	0.261	None	3
DTXSID1021871	4-Chlorophenol	239	0.029	0.250	None	2
DTXSID9034997	Tributyltetradecylphosphonium chloride	373	0.065	0.237	R8	2
DTXSID5020154	Clorophene	462	0.020	0.237	None	3

Six of the 11 chemicals have an antagonist prediction in the full ER pathway model presented in Judson et al. (2015).

Given the prevalence of antagonist predictions in the chemicals with a best 4-assay subset ER agonist model AUC above 0.214, we explored the chemical clusters containing 1) at least two ER model chemicals and 2) at least one false-positive chemical with an antagonist assignment from the Judson et al. (2015) ER model. Nine clusters met these criteria, covering a total of 62 chemicals including 11 false-positive chemicals with an antagonist assignment (Figure 1B). Next, we examined each of these nine clusters in more detail (Supplementary Data S3). This included creating heat maps that illustrated 1) which chemical in the cluster had a false-positive prediction, 2) the prediction from the Judson et al. (2015) ER model, and 3) the chemical-assay AUC values across the 16 assays present in the ER agonist pathway.

**FIGURE 4:**
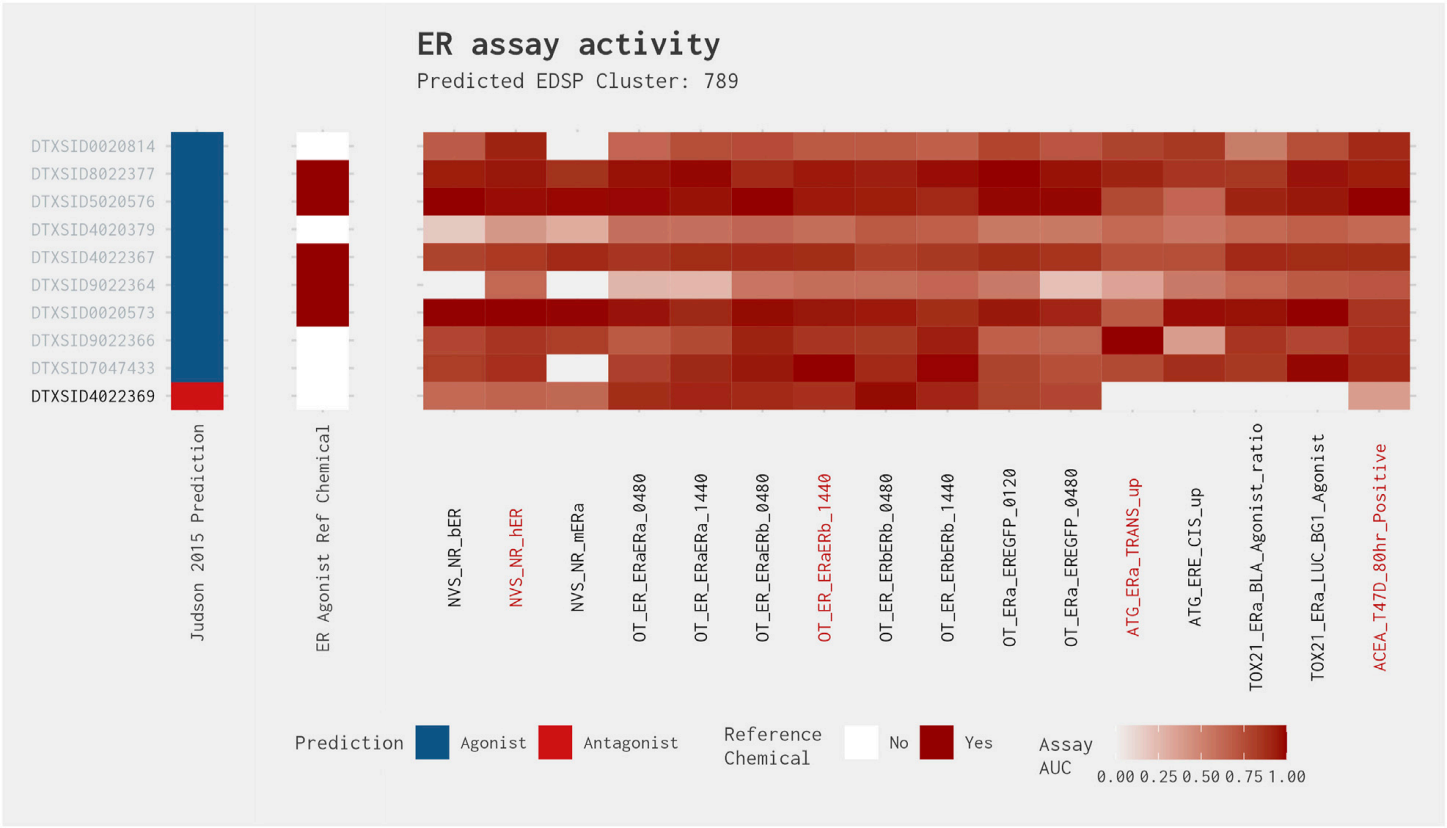
Illustration of the results for the ER agonist model chemicals in Cluster 789. The gray DTXSIDs represent chemicals correctly predicted by the best 4-assay subset ER agonist model compared with the full ER agonist model. The Judson et al. (2015) prediction heat map displays the results from the 2015 full ER pathway model. The middle heat map identifies whether the chemical is a reference chemical for ER agonism. The main heat map shows the AUC values for each chemical-assay pair with darker red representing larger AUC values (i.e., greater activity). The red assay labels represent the assays present in the best 4-assay ER agonist model. Chemical names associated with the DTXSIDs can be found in Supplementary Data S1.

Figure 4 illustrates the heat map for Cluster 789. This cluster comprises 10 chemicals, including known ER agonists, such as 17α-estradiol and 17α-ethinylestradiol. Nine of the chemicals in this cluster were correctly predicted when comparing the best 4-assay subset ER agonist model with the full ER agonist model (as shown by the gray text in Figure 4). Additionally, each of these chemicals was predicted to be an ER agonist by the Judson et al. (2015) ER model. Meanwhile, the one false-positive chemical in this cluster, fulvestrant, is a known ER antagonist and is predicted as such by the full ER pathway model (Judson et al., 2015). Comparing the activity of fulvestrant across all 16 assays present in the full ER agonist model against that of the other chemicals in the cluster, we can see a clear difference between the predicted agonists and antagonist. Although the agonists have relatively high activity across most, if not all, of the 16 assays, fulvestrant has high activity in those assays that overlap between the agonist and antagonist pathways and little to no activity in the assays that are ER agonist pathway specific, which is what would be expected from an ER antagonist.

This pattern of (relatively) high activity in the overlapping assays present in both agonist and antagonist pathways and little to no activity in the assays that are ER agonist pathway specific is also observed for the other three clusters with a best 4-assay subset ER agonist model AUC >0.214: Clusters 184, 419, and 534. However, the remaining five clusters that do not have a best 4-assay subset ER agonist model AUC >0.214 (i.e., Clusters 130, 247, 370, 592, and 621) do not exhibit the same pattern and, typically, have activity in relatively few assays. Although the false-positive chemicals present in Clusters 184, 419, 534, and 789 were predicted as false positives by the best 4-assay subset ER agonist model, this is likely only because potential antagonist effects were not being evaluated; thus, the chemicals could not be moved into the antagonist mode.

Therefore, including ER antagonist-related assays when screening potential ER-modulating chemicals would not only identify ER antagonists but should also improve the predictions regarding ER agonism. Within this strategy, one would initially test all chemicals of interest in a preliminary 6-assay battery consisting of four assays comparable with the best 4-assay subset ER agonist model and two ER antagonist-specific assays. One would then use the best 4-assay subset ER agonist model to calculate an ER agonist AUC for each chemical. Additionally, the data from the 6-assay battery could be used to create and validate a model to calculate an AUC for potential ER antagonism. Because the false-positive chemicals with an antagonist prediction would be expected to be identified as ER antagonists via the use of one or two additional assays, they were removed from further investigation. In addition, from the perspective of prioritization for testing, whether the chemical is identified as an agonist or an antagonist is less important than the fact that it has the potential to be an estrogen-active chemical.

**FIGURE 5:**
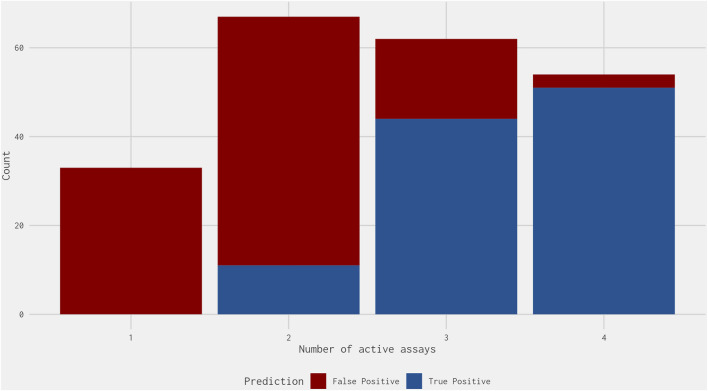
Stacked bar graph illustrating the count of the number of assays within the best 4-assay subset ER agonist model in which the true-positive (blue) and false-positive (red) chemicals were active. The false-positive chemicals do not include those predicted to be antagonists within the full ER pathway model results from Judson et al. (2015). Of the true positives, all but 11 chemicals were active in three or more assays. Meanwhile, of the false positives, all but 21 chemicals were active in two or fewer assays.

Next, we calculated the number of assays in the best 4-assay subset ER agonist model with activity for each false-positive chemical and compared this against the activity of the true-positive chemicals: activity was considered as a chemical-assay AUC value above 0. Most false-positive chemicals were active in one or two assays, with 18 (14.7%) false-positive chemicals active in three assays and only 3 (2.5%) active in four assays (Figure 5). Meanwhile, most true-positive chemicals were active in three or four assays, with no true-positive chemicals active in only one assay and only 11 (10.4%) active in two assays (Figure 5). Therefore, requiring a chemical to be active in three or four of the best 4-assay subset ER agonist model assays would remove 89 (80.9%) of the remaining 110 false-positive chemicals while only missing 11 true-positive chemicals (Figure 1B). Most of the 11 true-positive chemicals missed could be considered marginal actives, because 9 of the 11 have an AUC in the subset model between 0.1 and 0.2. This step compensates for the loss of the pseudoreceptor components in the original 16-assay model and could represent an additional criterion that may be of use for prioritization, with chemicals active in three or four assays being given a marginally higher priority than chemicals with a similar best 4-assay subset ER agonist model AUC active in only one or two assays. Although this would deprioritize 11 chemicals with a true-positive prediction, over 85% of the chemicals that meet this criterion are those with a false-positive prediction.

**FIGURE 6:**
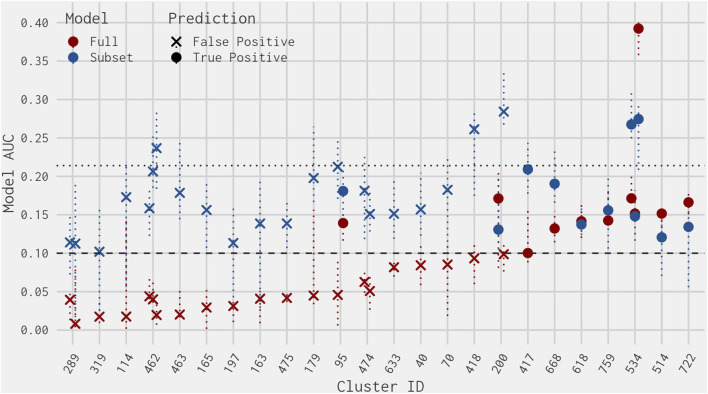
Jitter plot of AUC values from the best 4-assay subset ER agonist model (blue) and the full ER agonist model (red) for each of the 11 true-positive chemicals active in two assays (circles) and the 21 false-positive chemicals active in three or four assays not predicted to be an antagonist in the full ER pathway model from Judson et al. (2015) (crosses). Each cluster is separated by the predicted chemical Cluster ID. The dotted horizontal line at 0.1 represents the threshold for an ER agonist prediction. The dotted horizontal line at 0.214 represents the lower quartile of the best 4-assay subset AUC values of the true-positive chemicals. The vertical dotted lines represent the upper and lower 95% confidence intervals of the AUC predictions.

The names of the true-positive chemicals active in two assays and the false positives active in three or four assays in the best 4-assay subset ER agonist model can be found in Supplementary Table S1. Plotting the AUC values (including the upper and lower 95% confidence intervals) for the best 4-assay subset ER agonist model and full ER agonist model for these 32 chemicals, one can see that the majority have a best 4-assay subset ER agonist model AUC that is below the lower-quartile best 4-assay subset ER agonist model AUC of all true-positive chemicals (Figure 6). For several of the false-positive chemicals, the full ER agonist model AUC is near the cutoff for defining ER activity. Additionally, for other false-positive chemicals, the upper 95% confidence interval of the full ER agonist model AUC is close to or above the 0.1 cutoff (Watt and Judson, 2018). This suggests that the activity calls from the full ER agonist model are less certain for these chemicals. The same logic holds for true-positive predictions from the best 4-assay subset ER agonist model; the lower 95% confidence interval for several of these chemicals is close to or below the 0.1 threshold for defining ER agonist activity.

After further investigation of the true-positive chemicals with activity in two assays, we found that 91% were active in the ACEA assay and 73% were active in the ATG_ERa_TRANS_up assay. Only two chemicals (melengestrol acetate [DTXSID5048184] and 4-[hexyloxy]phenol [DTXSID4048195]) were active in the NovaScreen (NVS) receptor-binding assay. According to Judson et al. (2017), the NVS assays may miss many chemicals that only weakly bind ER. As such, although it is difficult to ascertain whether the inactivity observed in the NVS assay for the chemicals with a true-positive prediction is “real,” several have an “active (weak)” or “active (very weak)” binding prediction from the Collaborative Estrogen Receptor Activity Prediction Project (CERAPP) model (Mansouri et al., 2016). Some of these substances (such as rotenone [DTXSID6021248]), may act via estrogen-related receptor alpha (ERRα; NR3B1) (Lynch et al., 2019), which is not assessed as part of the EDSP Tier 1 battery of assays or the ToxCast ER Bioactivity Model (Judson et al., 2015).

Of the true positives active in two assays and false positives active in three or four assays in the best 4-assay subset ER agonist model, only six clusters (Clusters 289, 462, 95, 474, 200, and 534) contain more than one chemical (Figure 6 and Supplementary Table S1). These clusters were examined more closely to determine the possible reasons behind the differences between the best 4-assay subset ER agonist model and the full ER agonist model. Both chemicals from Cluster 289 were just above the threshold in the subset model and had low AUC values in the full model, so they are assumed to be truly false positives, which cannot be avoided in an initial screening assay. The remaining clusters are considered in more detail as follows.

**FIGURE 7:**
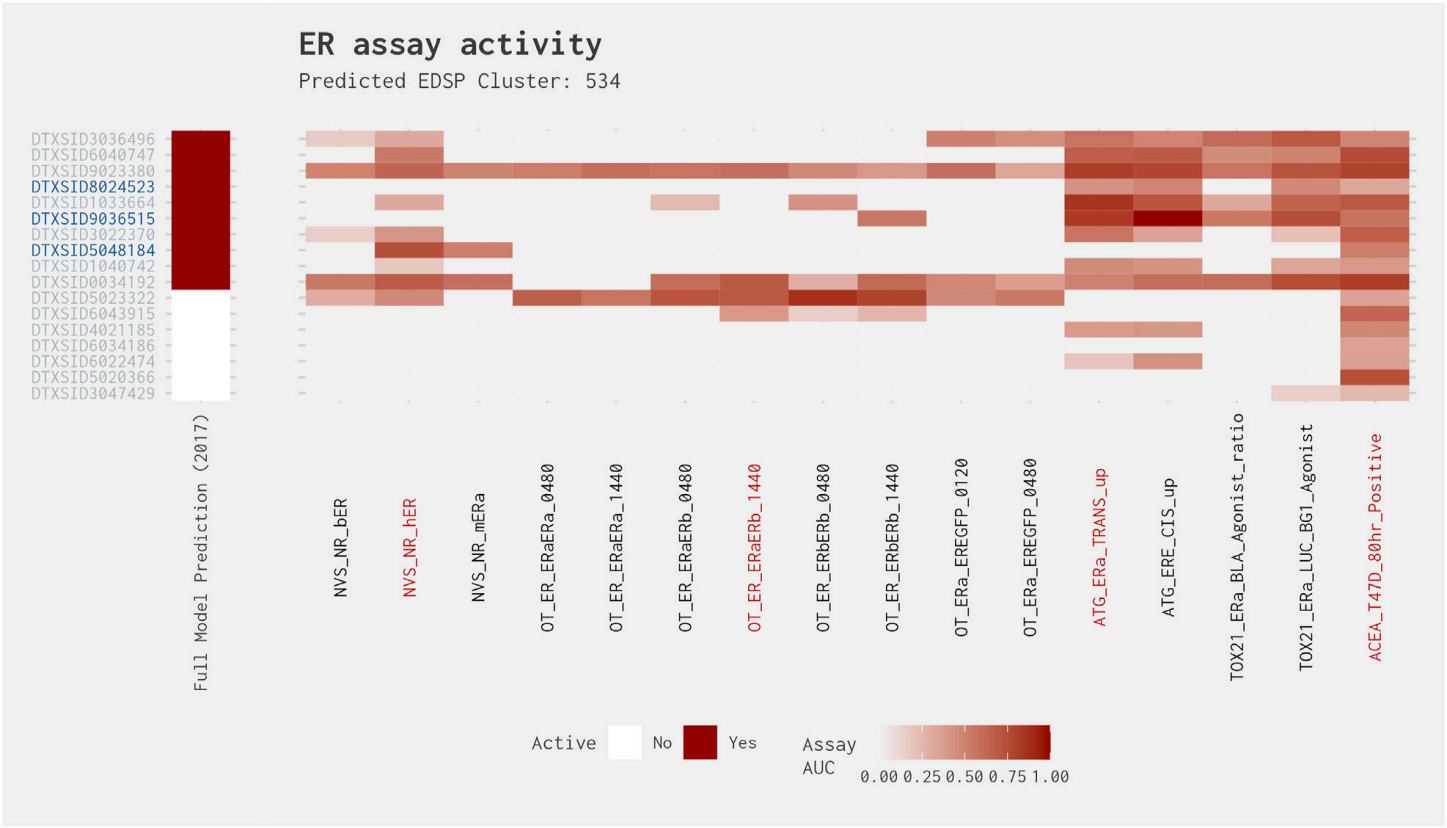
Illustration of the results for the ER agonist model chemicals in Cluster 534. Blue DTXSIDs represent true-positive chemicals active in two of the best 4-assay ER agonist subset model assays, orange DTXSIDs represent false-positive chemicals active in three or four of the best 4-assay ER agonist subset model assays, and gray DTXSIDs represent chemicals that do not meet these criteria. The “Full Model Prediction (2017)” heat map illustrates the results from the Judson et al. (2017) full ER agonist model, with red indicating chemicals predicted to be active and white indicating chemicals predicted to be inactive. The main heat map shows the AUC values for each chemical-assay pair with darker red representing larger AUC values.

Cluster 534 is the only cluster to have a true-positive chemical where the AUC in the subset model is greater than 0.214, but the activity is restricted to two assays. In fact, two of the three chemicals that were positive in the subset model and had activity in two assays (testosterone propionate [DTXSID9036515] and melengesterol acetate [DTXSID5048184]) had an AUC in the best 4-assay subset ER agonist model greater than 0.214. Cluster 534 is composed of 17 steroidal androgens, progestins, and/or glucocorticoids. Example chemicals within this cluster include prednisone (DTXSID4021185), corticosterone (DTXSID6022474), and 17-methyltestosterone (DTXSID1033664, an ER reference agonist). As can be seen in Figure 7, most chemicals in this cluster tend not to have activity in the eight Odyssey Thera (OT) assays evaluating biological process targets of protein stabilization and gene expression (Judson et al., 2015; Judson et al., 2017). Of the four assays present in the best 4-assay subset ER agonist model, both testosterone propionate and the remaining true positive with activity in two assays (4-androstene-3,17-dione [DTXSID8024523]) were active in the ATG transactivation assay (ATG_ERa_TRANS_up) and the ACEA cell proliferation assay (ACEA_T47D_80h_Positive). Both chemicals were most active in the ATG transactivation assay. It was pointed out by Judson et al. (2015) that this transactivation assay is highly multiplexed, with concurrent androgen receptor and ER readouts, along with some metabolic capability. Therefore, the observed activity of these chemicals may be “real” estrogenic activity, due to metabolic activation of the chemicals, or it may be being caused by cross-reactivity due to the assay technology. Melengestrol acetate, meanwhile, was quite highly active in the NVS receptor binding (NVS_NR_hER) and ACEA cell proliferation assays, with assay-specific AUCs of 0.735 and 0.521, respectively. Relatively high concentrations of melengestrol acetate have previously been reported to be able to bind to the ER and induce cell proliferation (Le Guevel and Pakdel, 2001; Perry et al., 2005). Furthermore, the T47D cell line used within the ACEA cell proliferation assay is known to be highly responsive to progestins and glucocorticoids (Capen and Martin, 1989; Judson et al., 2015). It appears that in addition to their primary nuclear receptor target, these chemicals may also exhibit (weak) ER agonism.

**FIGURE 8:**
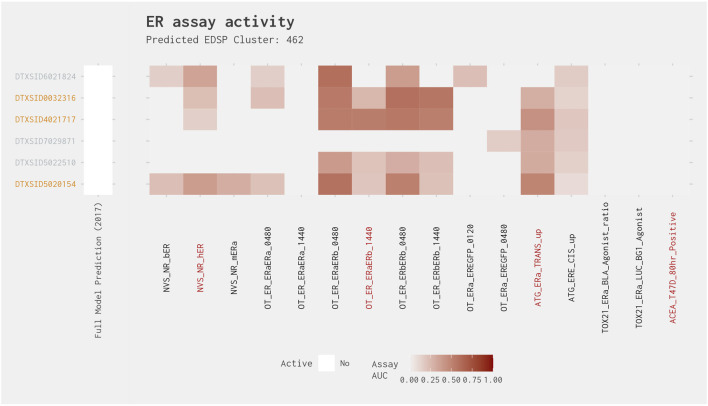
Illustration of the results for the ER agonist model chemicals in Cluster 462. Blue DTXSIDs represent true-positive chemicals active in two of the best 4-assay ER agonist subset model assays, orange DTXSIDs represent false-positive chemicals active in three or four of the best 4-assay ER agonist subset model assays, and gray DTXSIDs represent chemicals that do not meet these criteria. The “Full Model Prediction (2017)” heat map illustrates the results from the Judson et al. (2017) full ER agonist model, with red indicating chemicals predicted to be active and white indicating chemicals predicted to be inactive. The main heat map shows the AUC values for each chemical-assay pair with darker red representing larger AUC values.

Cluster 462 contained six chemicals, all of which were predicted to be negative in the full model. Three of these six chemicals were predicted to be positive in the subset model, with one chemical (clorophene [DTXSID5020154]) having an AUC from the subset model greater than 0.214, whereas the remaining two chemicals (4-chloro-3-methylphenol [DTXSID4021717] and 4-chloro-3,5-dimethylphenol [DTXSID0032316]) had a subset model AUC less than 0.214 (Figure 6). All subset model–positive chemicals had activity in three of the four assays and would, therefore, be included if the two active assay filter is applied. There is relatively consistent activity across the cluster, with all chemicals active in multiple OT protein stabilization assays and/or ATG assays (Figure 8). The greatest activity was observed in the OT_ER_ERaERb and OT_ER_ERbERb assays for all chemicals, except clotrimazole (DTXSID7029871). Because of the consistency in activity, we investigated the best 4-assay subset ER agonist model AUC values of the chemicals predicted to be negative in both the full and subset ER agonist models and discovered that all best 4-assay subset ER agonist model AUC values were ≥0.07: two chemicals (dichlorophen [DTXSID6021824] and 4-chloro-2-methylphenol [DTXSID5022510]) had a subset model AUC >0.09, which is very close to the 0.1 threshold. Further investigation of the best 4-assay subset ER agonist model active chemicals revealed that clorophene and 4-chloro-3-methylphenol have been identified as possibly having some slight ER activity, especially *in vitro*, where these chemicals have been observed to weakly bind ER (Körner et al., 1998; ECHA, 2017; Kenda et al., 2020). Moreover, 4-chloro-2-methylphenol has been seen to bind very weakly to ER, with a relative binding affinity of 0.00021% compared with 17β-estradiol (Körner et al., 1998; Blair et al., 2000). Therefore, it appears likely that these chemicals may have the potential to all be very weak ER agonists and, as such, may be close to the limit of detection for the assays.

**FIGURE 9:**
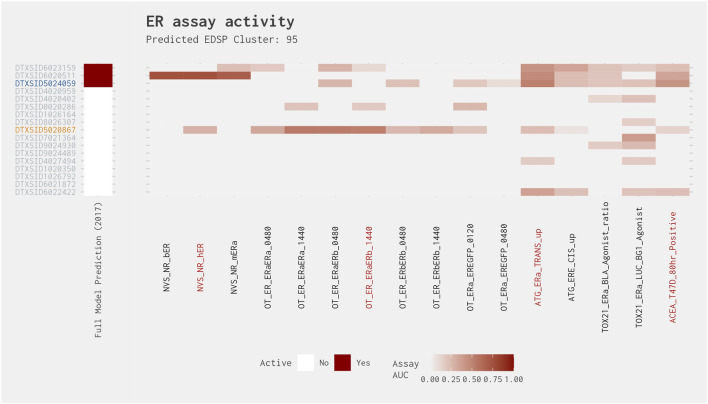
Illustration of the results for the ER agonist model chemicals in Cluster 95. Blue DTXSIDs represent true-positive chemicals active in two of the best 4-assay ER agonist subset model assays, orange DTXSIDs represent false-positive chemicals active in three or four of the best 4-assay ER agonist subset model assays, and gray DTXSIDs represent chemicals that do not meet these criteria. The “Full Model Prediction (2017)” heat map illustrates the results from the Judson et al. (2017) full ER agonist model, with red indicating chemicals predicted to be active and white indicating chemicals predicted to be inactive. The main heat map shows the AUC values for each chemical-assay pair with darker red representing larger AUC values.

Clusters 95 and 200 have chemicals that are positive in both the full and subset models but have activity in only two assays from the best 4-assay subset ER agonist model. They also have chemicals that are positive in the best 4-assay subset ER agonist model but with no activity in the full ER agonist model. With one exception, all AUCs from both models were less than 0.21 (Figure 6). Cluster 95 showed spotty activity across all assays (Figure 9). Both the full ER agonist model and the best 4-assay subset ER agonist model predicted two positives from this cluster. Both models predicted 3,3′-dimethylbenzidine dihydrochloride (DTXSID6020511) to be positive regardless of filters. The best 4-assay subset ER agonist model also predicted 3,3′-dimethylbenzidine (DTXSID5024059) to be positive, but this chemical showed activity in only two of the four assays, so it would be excluded if that filter is applied. In addition, the best 4-assay subset ER agonist model picked up 4,4′-diamino-3,3′-dimethyldiphenylmethane (DTXSID5020867), which has an AUC value of only 0.0458 in the full ER agonist model. Visually examining the results from the individual assays, however, it is impossible to definitively establish a difference among these chemicals with regard to the potential for estrogenic activity (Figure 9). It is unclear whether the chemical properties of this cluster are unsuitable for HT screening, which could cause variability across the assays, or whether this cluster contains chemicals that are extremely weak agonists and therefore fall at the limit of detection for the assays. Regardless of the reason, these chemicals are likely all weak agonists or all false positives, and the best 4-assay subset ER agonist model would flag this cluster as effectively as the full ER agonist model does.

**FIGURE 10:**
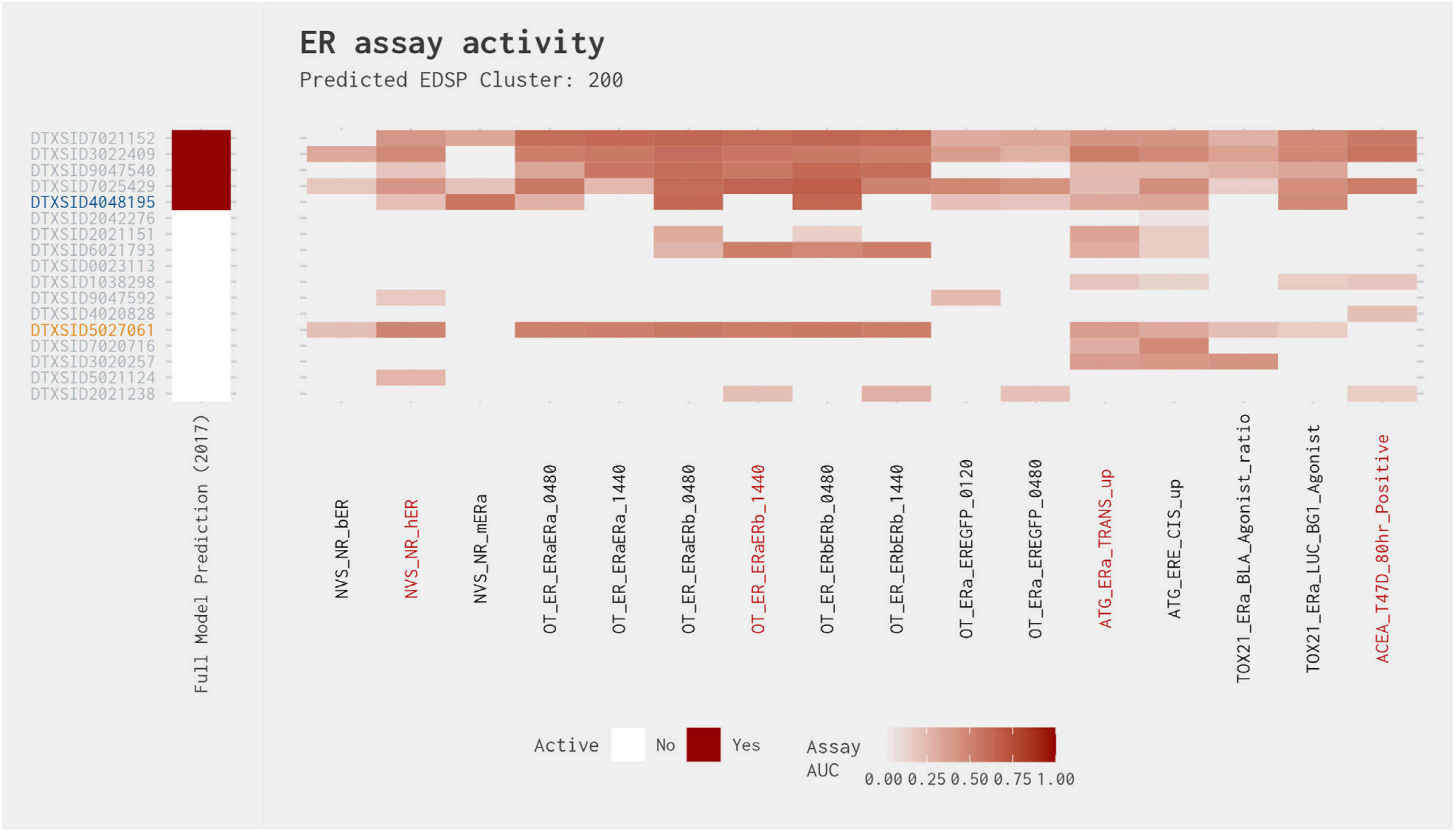
Illustration of the results for the ER agonist model chemicals in Cluster 200. Blue DTXSIDs represent true-positive chemicals active in two of the best 4-assay ER agonist subset model assays, orange DTXSIDs represent false-positive chemicals active in three or four of the best 4-assay ER agonist subset model assays, and gray DTXSIDs represent chemicals that do not meet these criteria. The “Full Model Prediction (2017)” heat map illustrates the results from the Judson et al. (2017) full ER agonist model, with red indicating chemicals predicted to be active and white indicating chemicals predicted to be inactive. The main heat map shows the AUC values for each chemical-assay pair with darker red representing larger AUC values.

Cluster 200 shows a pattern similar to that of Cluster 95, except for four chemicals that show consistent activity across most of the assays and are therefore called active in both models (Figure 10). Excluding those chemicals, however, we have the same situation where the full ER agonist model predicts two positive chemicals (4-[hexyloxy]phenol [DTXSID4048195] and 3-hydroxyfluorene [DTXSID9047540]) and the best 4-assay subset ER agonist model predicts two positive chemicals (2-naphthalenol [DTXSID5027061] and 3-hydroxyfluorene [DTXSID9047540]) with one in common and two discrepancies. The consistent activity for three chemicals from this cluster would suggest that the other chemicals are likely agonists as well. However, from the standpoint of screening chemicals, the best 4-assay subset ER agonist model is just as likely to flag chemicals from this cluster for further evaluation as the full ER agonist model. As additional data are gathered for the chemicals that are predicted to be agonists, structure-based predictions should assist with prioritizing the other chemicals within the cluster where the results are currently equivocal.

**FIGURE 11:**
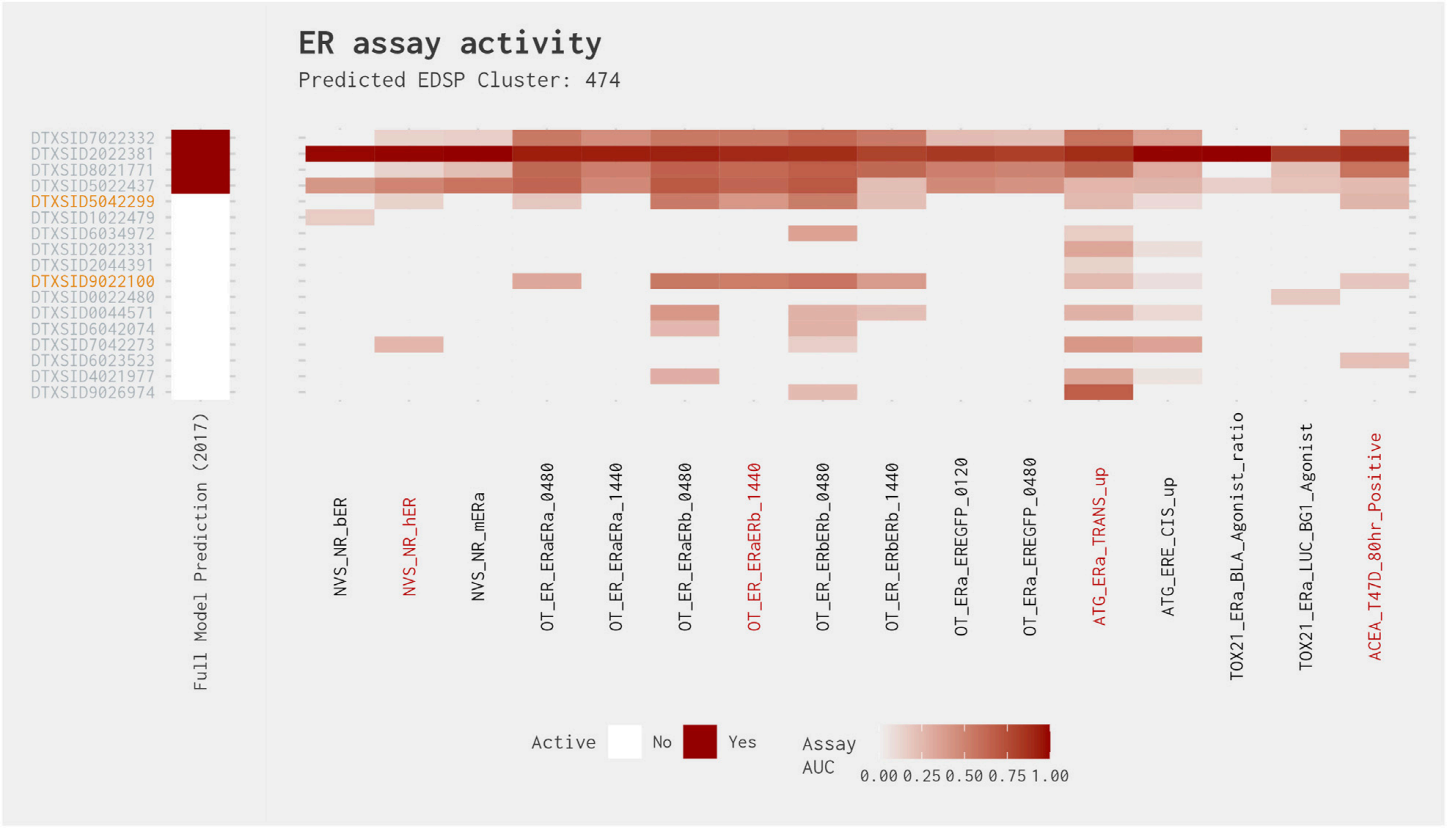
Illustration of the results for the ER agonist model chemicals in Cluster 474. Blue DTXSIDs represent true-positive chemicals active in two of the best 4-assay ER agonist subset model assays, orange DTXSIDs represent false-positive chemicals active in three or four of the best 4-assay ER agonist subset model assays, and gray DTXSIDs represent chemicals that do not meet these criteria. The “Full Model Prediction (2017)” heat map illustrates the results from the Judson et al. (2017) full ER agonist model, with red indicating chemicals predicted to be active and white indicating chemicals predicted to be inactive. The main heat map shows the AUC values for each chemical-assay pair with darker red representing larger AUC values.

Similar to Cluster 200, Cluster 474 contains a set of four chemicals that exhibit relatively high activity across most of the ER assays and are predicted active in both the full ER agonist model and the best 4-assay subset ER agonist model (Figure 11). However, in this instance, all chemicals predicted to be active in both models are active in all four of the best 4-assay subset ER agonist model assays. Excluding these chemicals, the best 4-assay subset ER agonist model predicts four positive chemicals (2,4-di-tert-pentylphenol [DTXSID9026974], 2,4-diisopropylphenol [DTXSID7042273], 4-propylphenol [DTXSID9022100], and 4-isopropylphenol [DTXSID5042299]). Two of these four chemicals are active in three (4-propylphenol [DTXSID9022100]) or all four (4-isopropylphenol [DTXSID5042299]) of the best 4-assay subset ER agonist model assays. Again, this consistent activity suggests that the other chemicals in this cluster could also be agonists. However, given the relatively large proportion of chemicals that exhibit activity mainly in the Attagene assays, there may be an activity cliff whereby some of the chemicals in this cluster may elicit activity via the associated pseudoreceptor, which may result in this cluster being split in two. As with Cluster 200, additional data generated for the chemicals predicted to be agonists should help to prioritize the remaining chemicals in the cluster.

### 3.6 Implications for a multi-stage prioritization workflow prior to EDSP tiered testing

**FIGURE 12:**
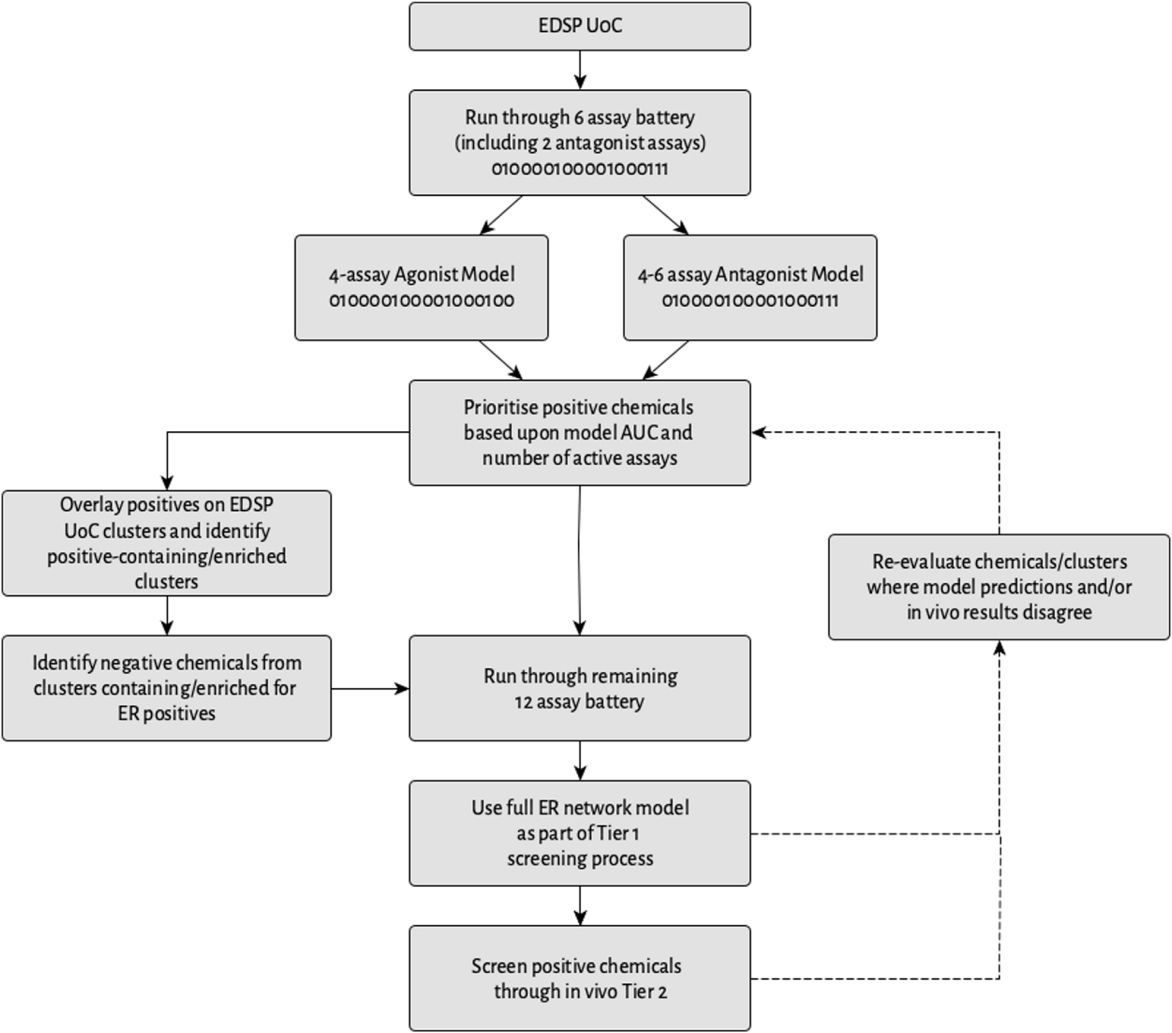
Workflow of a potential multi-stage screening strategy. The 0/1 coding in certain boxes represents assays from the full ER pathway model in which chemicals would (1) or would not (0) be screened.

Everything considered, one potential multi-stage prioritization workflow that could be implemented to assist in identifying potential endocrine disruptors acting via ER is illustrated in Figure 12. As part of this, the EDSP UoC would be tested in an initial battery of six assays composed of four assays comparable with the best 4-assay subset ER agonist model, as well as two antagonist-specific assays. These data would then be used as input to the best 4-assay subset ER agonist model, which would be utilized to calculate an ER agonist AUC for each chemical. Meanwhile, the creation and validation of an ER antagonist model utilizing data either from all six of the assays in the battery, or a subset thereof, should enable the calculation of an ER antagonist AUC. Any chemical with a positive prediction from either the ER agonist or ER antagonist model would be prioritized for Tier 1 screening.

The potential short-term impact of data collected using the 6-assay battery over a 4-assay battery comparable with the best 4-assay subset ER agonist model may be minimal, because, as mentioned in Section 3.5, in terms of prioritizing chemicals for testing, identifying a chemical as a potential estrogen-active is more important than identifying whether it is an agonist or antagonist. However, in the longer-term, the potential benefits of collecting these additional data include being able to use the additional data from the antagonist-specific assays to help distinguish agonists from antagonists, which should, in turn, allow for a more comprehensive identification of both agonists and antagonists.

The results from this study suggest that the positive chemicals could be prioritized based on a combination of the model AUC and the number of active assays. Subsequently, the data from the initial screening could be overlaid onto the EDSP UoC clusters and used to identify clusters that either contain ER-active chemicals or are enriched for ER activity. From here, positive chemicals, as well as chemicals with a negative prediction present within clusters containing or enriched for ER activity, would then be screened through the remaining 12 ER assays and the full ER pathway model used to re-calculate the ER agonist and antagonist AUC values. The results from this model could then be used as an alternative to conducting the ER binding *in vitro*, ER transcriptional activation *in vitro*, and rat uterotrophic assays (U.S. EPA, 2023). Chemicals with a positive prediction in the full ER pathway model would be prioritized for Tier 2 testing, with the AUC values from the full ER pathway model and whether an agonist or antagonist prediction was stronger than that of the pseudoreceptors being used as criteria to set the prioritization.

Additionally, where the results from the full ER network model and the initial assay battery model disagree, the chemicals and/or clusters can be re-evaluated. Further re-evaluation also could be conducted for those chemicals and/or clusters where the model predictions disagree with the Tier 2 *in vivo* results. As more and more data are generated by the screening workflow and tiered testing, these data have the potential to be further utilized for prioritization or other purposes. Examples include the identification of pharmacophores or toxicophores associated with potential endocrine effects via ER, or the prioritization of clusters that contain large proportions of chemicals active across many assays but that may not quite surpass the 0.1 threshold because these chemicals may still be of concern, among other potential uses.

Although there are assays in the current model that no longer exist, this information is provided as a proof of concept that can still be informative for evaluating subset models and developing prioritization and screening plans for future models.

## 4 Conclusion

We separated the EDSP UoC into 826 clusters based upon structural similarity among the chemicals. The chemicals used to build the full ER agonist model and subsequent best 4-assay subset ER agonist model provide relatively good coverage of the EDSP UoC, with 1,730 ER model chemicals mapping to 557 EDSP UoC clusters (67%). As desired for an early-stage test, the sensitivity was particularly high, with 122 of 124 discrepancies being a false-positive call from the best 4-assay subset ER agonist model compared with the full ER agonist model. When considering implications for chemical screening and prioritization, it is important to note that the results from the best 4-assay subset and 16-assay full ER agonist models agree 87.8% of the time for stronger agonists’ calls (i.e., when the AUC for the agonist model was 0.214 or greater, which corresponds to the lower 25th percentile of the active chemicals based on the best 4-assay subset ER agonist model). The performance of the model seemed to be independent of the chemical structure with 122 false positives distributed across 84 clusters, with most cases being a single false positive per cluster.

Because more than 90% of the false positives have an AUC below the lower 25th percentile, they would be unlikely to be prioritized for screening in the short term. As additional screening is done, the model predictions should improve as our understanding of the structural features and bioactivity measurements that are most predictive improves. In addition, 10% of the false-positive agonist predictions (representing over half of the false positives with an AUC above the lower 25th percentile) are predicted to be antagonists by the full ER pathway model. Because subsequent testing would distinguish between agonism and antagonism, any activity prediction at this stage would be equally valuable for prioritizing those chemicals to undergo future screening. Of the false positives that are not predicted to be antagonists by the full ER pathway model, over 80% can be filtered by requiring activity in at least three of the four assays in the best 4-assay subset ER agonist model, which only removes ∼10% of the true-positive chemicals.

Many potential false-positive predictions fall within clusters with other chemicals predicted to be weak ER agonists by the full ER agonist model and, therefore, could as easily represent a false-negative call by the full ER agonist model instead of a false-positive call by the best 4-assay subset ER agonist model. Incorporation of antagonist assays along with the four assays corresponding to the best 4-assay subset ER agonist model could help distinguish antagonists if desired, and additional data collected when screening the higher-priority chemicals should allow a combination of *in silico* and *in vitro* predictions to better distinguish weak agonists from inactive chemicals.

Although this is a descriptive analysis of previous results, several lessons learned could be applied to any testing battery used in the future. First, the clustering of the chemicals provides a means of ensuring that future testing covers the full chemical space represented within the EDSP UoC. The clusters can also assist in prioritizing chemicals with a weak signal in a future 4-assay (or other minimal assay configuration) battery. A chemical with a marginal signal that falls within a cluster with other known agonists would be a higher priority for future testing than one that falls within a cluster with known inactive chemicals. The incorporation of the filter for activity in more than 50% of the assays when utilizing a minimal assay model is also a reasonable criterion when trying to minimize false positives.

Furthermore, this workflow can be applied to future datasets as they are generated by leveraging the clusters to borrow information from the 1,730 chemicals tested in the full ER model. As new chemicals are tested, read-across analysis can be used to estimate their activity in the full ER model, which can then be compared with the measured values from the new assay battery and associated model. Because any new assays are likely to be validated by comparing the results from chemicals from the original chemical list in the new assay, this information can also be used to translate findings from any future assay battery back to the original dataset. As additional chemicals are prioritized for testing in the full ER battery, the workflow can be repeated using the measured data for chemicals from the minimal assay battery compared against the new full assay battery.

As more data are collected, the *in silico* models should improve and decrease the reliance on bioactivity alone. Here again, the clustering approach can be useful in providing a framework to evaluate which portions of the EDSP UoC chemical space are reliably covered by *in silico* and *in vitro* approaches and where predictions from either method alone or both methods combined are most reliable. Although the clusters generated by using features representing the overall chemical structure will not always capture the functionally relevant structural features that drive receptor binding or activation potential, they allow the EDSP UoC to be separated into smaller units, which can then be investigated in depth, as illustrated for the clusters highlighted in this article.

In conclusion, this case study presents a proof of concept for evaluating subset models and providing support for their use in screening and prioritization strategies. The high sensitivity demonstrated in this case study is desirable to avoid filtering chemicals too early. Chemicals of highest priority are consistently identified by both models, and simple rules can be applied that distinguish chemicals likely to be positive from those without support from the full ER agonist model. The lessons learned from this case study can easily be applied to future testing regardless of whether the same assays are used, and this general workflow can be applied to future datasets (either independently or after harmonizing and merging with the previous data) to understand the performance of the models associated with the testing battery.

## Data Availability

The original contributions presented in the study are included in the article/Supplementary Material, further inquiries can be directed to the corresponding author.

## Footnotes

1 The file is available at https://www.epa.gov/sites/default/files/2015-07/documents/edsp_chemical_universe_list_11_12.pdf.

2 Between the results from Judson (2015) and invitrodb v3.3, there are five chemicals whose CASRNs differ and two ER assays whose names have changed. These are identified in the check “casrn_from_judson_2015_to_ivdb_v3-3.R″ and “data_setup_tidyverse.R″ scripts.
