# Supplementary material for: Chemistry domain of applicability evaluation against existing estrogen receptor high-throughput assay-based activity models
Source: Front Toxicol. 2024 Apr 17;6:1346767. doi: 10.3389/ftox.2024.1346767 (PMC11061348; doi:10.3389/ftox.2024.1346767)
Supplement: Supplementary file 1 [file Table1.docx]

Supplementary Material

Chemistry domain of applicability evaluation for existing estrogen receptor high-throughput assay-based activity models

Mark D. Nelms, Todor Antonijevic, Caroline Ring, Danni Harris, Ronnie Joe Bever*, Scott G. Lynn, David Williams, Grace Chappell, Rebecca Boyles, Susan Borghoff, Stephen W. Edwards, Kristan Markey

*** Correspondence:** Ronnie Joe Bever: [Bever.Ronnie@epa.gov](mailto:Bever.Ronnie@epa.gov)

# Supplementary Data

Supplementary Data S1. Csv file containing chemical identifiers (i.e., DTXSID, DTXCID, chemical name, CASRN), structural information, and cluster identifier for each chemical (and chemical component) in the EDSP UoC for which a SMILES string could be retrieved from the CompTox Chemicals Dashboard.

Supplementary Data S2. Csv file containing chemical identifiers (i.e., DTXSID, DTXCID, chemical name, CASRN), structural information, and the cluster identifier for each ToxCast chemical for which a SMILES string could be retrieved from the CompTox Chemicals Dashboard. The csv file also contains columns indicating whether the is in the EDSP UoC or in the ER model chemical list from Judson et al. (2015).

Supplementary Data S3. Csv file containing cluster information and results from the full ER agonist model, best 4-assay ER agonist subset model, and the full ER pathway model for chemicals in clusters containing at least one false-positive chemical.

# Supplementary Figures and Tables

## Supplementary Figures


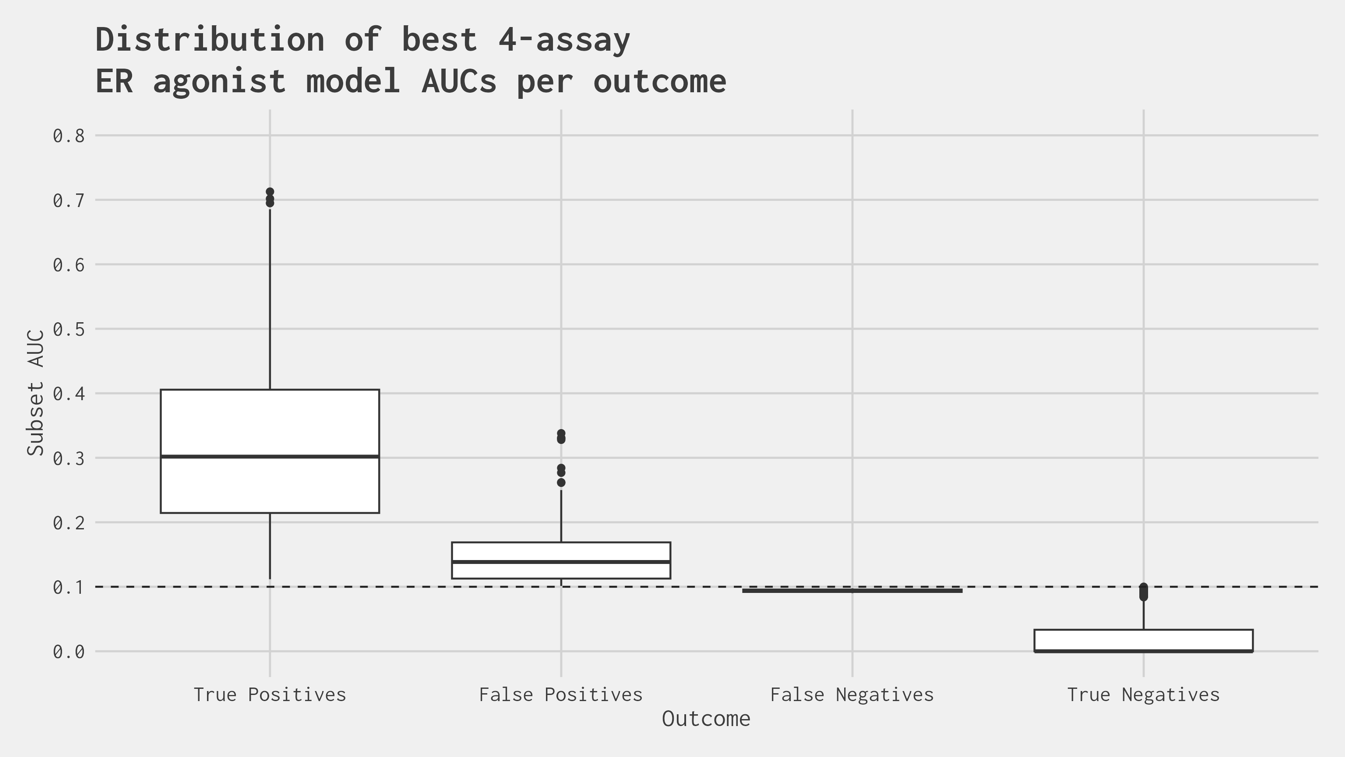


**Supplementary Figure S1.** Box and whisker plot illustrating the distribution of all AUC values for the best 4-assay subset ER agonist model per outcome, when compared against the full ER agonist model.

## Supplementary Tables

**Supplementary Table S1**. List of the 11 chemicals with a true positive prediction that are active in two of the four assays in the best 4-assay subset ER agonist model and the 21 chemicals with a false positive prediction that are active in three or four of the four assays in the best 4-assay subset ER agonist model.

| **DTXSID** | **Name** | **Outcome** | **Number Active Assays** | **Full model AUC**  **(95% CI)** | | **Subset model AUC**  **(95% CI)** |
| --- | --- | --- | --- | --- | --- | --- |
| DTXSID3021984 | 1-Dodecanamine | True Positive | 2 | 0.152 (0.125-0.155) | 0.121 (0.0703-0.144) | |
| DTXSID4048195 | 4-(Hexyloxy)phenol | True Positive | 2 | 0.171 (0.0808-0.207) | 0.131 (0.106-0.198) | |
| DTXSID1027633 | Irganox 1010 | True Positive | 2 | 0.166 (0.151-0.179) | 0.134 (0.0556-0.16) | |
| DTXSID1029170 | 4-Methylpent-3-en-2-one | True Positive | 2 | 0.142 (0.12-0.164) | 0.137 (0.124-0.159) | |
| DTXSID8024523 | 4-Androstene-3,17-dione | True Positive | 2 | 0.152 (0.124-0.175) | 0.148 (0.116-0.182) | |
| DTXSID7020685 | beta-Hexachlorocyclohexane | True Positive | 2 | 0.143 (0.137-0.179) | 0.156 (0.106-0.2) | |
| DTXSID5024059 | 3,3'-Dimethylbenzidine | True Positive | 2 | 0.139 (0.116-0.153) | 0.181 (0.149-0.211) | |
| DTXSID4021135 | C.I. Solvent Yellow 14 | True Positive | 2 | 0.132 (0.114-0.155) | 0.19 (0.166-0.233) | |
| DTXSID5032573 | Pyridaben | True Positive | 2 | 0.1 (0.088-0.157) | 0.209 (0.177-0.244) | |
| DTXSID5048184 | Melengestrol acetate | True Positive | 2 | 0.171 (0.165-0.203) | 0.268 (0.249-0.312) | |
| DTXSID9036515 | Testosterone propionate | True Positive | 2 | 0.392 (0.358-0.406) | 0.274 (0.208-0.294) | |
| DTXSID7020392 | Diallyl phthalate | False Positive | 3 | 0.0175 (0.00904-0.0424) | 0.102 (0.0166-0.161) | |
| DTXSID4034497 | Metconazole | False Positive | 3 | 0.00821 (0.00363-0.0832) | 0.113 (0.0168-0.19) | |
| DTXSID0021597 | Heptanal | False Positive | 3 | 0.0313 (0.0103-0.0373) | 0.113 (0.0426-0.16) | |
| DTXSID0032493 | Triadimenol | False Positive | 3 | 0.0394 (0.0129-0.0472) | 0.114 (0.0722-0.15) | |
| DTXSID2023430 | Pentaerythritol tetranitrate | False Positive | 3 | 0.0408 (0.00888-0.051) | 0.139 (0.0288-0.193) | |
| DTXSID7040788 | 2-tert-Butyl-4-methoxyphenol | False Positive | 3 | 0.0418 (0.0366-0.0496) | 0.139 (0.107-0.171) | |
| DTXSID5041691 | Butam | False Positive | 3 | 0.0817 (0.0694-0.0908) | 0.151 (0.119-0.194) | |
| DTXSID9022100 | 4-Propylphenol | False Positive | 3 | 0.0508 (0.0265-0.0732) | 0.151 (0.128-0.182) | |
| DTXSID6025436 | 8-Hydroxyquinoline sulfate | False Positive | 3 | 0.0294 (0.00147-0.0538) | 0.156 (0.0987-0.196) | |
| DTXSID6028022 | 1-(Bromomethyl)-3-phenoxybenzene | False Positive | 3 | 0.0843 (0.0585-0.11) | 0.157 (0.123-0.205) | |
| DTXSID0032316 | 4-Chloro-3,5-dimethylphenol | False Positive | 3 | 0.0439 (0.04-0.0717) | 0.158 (0.122-0.185) | |
| DTXSID5040708 | 4-Hexylaniline | False Positive | 3 | 0.0176 (0.00163-0.143) | 0.173 (0.0564-0.219) | |
| DTXSID4034576 | 2-Bromo-4-hydroxyacetophenone | False Positive | 3 | 0.0202 (0.0164-0.0557) | 0.179 (0.144-0.244) | |
| DTXSID1020699 | 4-Hexylresorcinol | False Positive | 3 | 0.0449 (0.0336-0.157) | 0.198 (0.167-0.265) | |
| DTXSID4021717 | 4-Chloro-3-methylphenol | False Positive | 3 | 0.04 (0.0354-0.0638) | 0.206 (0.185-0.258) | |
| DTXSID5020154 | Clorophene | False Positive | 3 | 0.0196 (0.0068-0.0392) | 0.237 (0.184-0.284) | |
| DTXSID3040776 | Morin hydrate | False Positive | 3 | 0.0933 (0.0597-0.111) | 0.261 (0.175-0.288) | |
| DTXSID5027061 | 2-Naphthalenol | False Positive | 3 | 0.0987 (0.0761-0.114) | 0.284 (0.267-0.336) | |
| DTXSID5042299 | 4-Isopropylphenol | False Positive | 4 | 0.0626 (0.0484-0.0801) | 0.181 (0.118-0.225) | |
| DTXSID2032558 | Kresoxim-methyl | False Positive | 4 | 0.0852 (0.0183-0.116) | 0.182 (0.107-0.226) | |
| DTXSID5020867 | 4,4'-Diamino-3,3'-dimethyldiphenylmethane | False Positive | 4 | 0.0458 (0.00587-0.0908) | 0.212 (0.171-0.251) | |
